# A scientometric study of *Maqasid al-shariah* research: trending issues, hotspot research, and co-citation analysis

**DOI:** 10.3389/frma.2024.1439407

**Published:** 2024-11-27

**Authors:** Tawffeek A. S. Mohammed

**Affiliations:** Department of Foreign Languages, University of the Western Cape, Cape Town, South Africa

**Keywords:** *maqāṣid al-shariī'ah*, research, scientometric, author keywords, co-citation, organizations

## Abstract

This study examines research on *maqāṣid al-shariī'ah* in journals indexed in Web of Science (WoS) and Scopus. As *maqāṣid al-shariī'ah* plays a vital role in guiding Islamic legal theory and contemporary applications of Islamic law in various sectors of life, familiarity with the scholarly landscape of the field is essential for assessing its growing influence in both academic and practical contexts. Hence, this study aims to explore the trajectory of research in *maqāṣid* studies, identify its key focus areas, and conduct a document co-citation analysis to uncover patterns in scholarly collaboration and influence. In addition, the study examines contributing countries, organizations, and leading journals in this field. Four Hundred documents published between 2000 and 2022 were retrieved and analyzed using the metrics functionalities of both databases. In addition, advanced analytical tools including Publish or Perish, VOSviewer, and ScientoPy v1.3.5 were utilized to conduct a multifaceted examination that encompasses document co-citation, sources co-citation, and authors' keyword analyses, among others. Data were carefully filtered to include research related to *maqāṣid al-shariī'ah* as an area of applied Islamic thought and its applications in different disciplines. The findings of the study revealed that research outputs in *maqāṣid al-shariī'ah* studies span various disciplines including religion, business and economics, science and technology, and medicine among others. The development of publications between 2000 and 2022 for the two databases indicates distinct upward trends in cumulative publications and annual growth. A vibrant and diverse global research landscape exists for *maqāṣid al-shariī'ah*, with Malaysia and Indonesia leading in terms of productivity and impact. This article presents original findings which may be of significance to researchers in Islamic studies, applied Islamic thought, and related interdisciplinary and multidisciplinary fields. This scientometric study is limited to English journal articles that were published between 2000 and 2022 on *maqasid* research in Scopus and Web of Science (WoS). Given the dynamic nature of these two databases, where results can fluctuate rapidly due to the continuous addition of new papers or the retraction of existing articles, this study is limited to the datasets that were created at the time of investigation. These limitations might influence the generalizability of the findings.

## 1 Introduction

Recently, numerous scientometric publications have been published in different academic disciplines. These studies seek to employ scientific methods and techniques to quantitatively examine scholarly publications to determine the prevailing focus of research in a specific discipline and to discern the scientific contributions and research outputs in the discipline (Carollo et al., 2021). A scientometric analysis is different from bibliometric analysis. Unlike the latter, scientometric analysis not only focuses on the quantitative or statistical aspects of scholarly publications, such as authors, institutions, countries, citation counts, publication metrics, and trends, but also tackles the qualitative aspects of scholarly publications, including the key themes and emerging trends of research in a particular field (Mohsen et al., 2023). The use of scientometric analysis enables researchers to gain valuable insights into the dynamics and development of a particular academic discipline. Hence, it may guide future research directions and policy decisions. Furthermore, scientometric analysis can also provide a broader understanding of the overall landscape of research in a particular academic discipline and identify gaps and areas that require further exploration or investigation. Scientometric analysis, at the time this study was conducted, appears to be absent in religious studies at large and in Islamic studies in particular. Bibliometric studies and systematic reviews in some areas of religious studies often rely on subjective selections of researchers and are predominantly qualitative in nature. There is a pressing need for more studies to explore the dynamics of certain branches of Islamic studies. This includes contemporary jurisprudence trends such as *maqāṣid al-shariī'ah* studies, *Fiqh al-Taḥawwulāt* “the jurisprudence of transitions”, and the jurisprudence of minorities, among others. As *maqāṣid al-shariī'ah* plays a critical role in guiding Islamic legal theory and modern applications of Islamic law, understanding its scholarly trajectory is essential for assessing its growing influence in both academic and practical contexts. The moderate school of Islamic thought, which this study explores, has significant applications across various sectors, including Islamic banking, the Halal industry, interfaith dialogue, biomedical ethics, and gender equality, to name a few. Scholars and students in Islamic studies, law, and related fields may find a scientometric study on *maqāṣid al-shariī'ah* highly valuable. It provides insights into the evolution, influence, and current trends in this specific area of research. Moreover, a scientometric study of this type can show intersections between Islamic thought and other fields like economics, law, ethics, and environmental studies, fostering interdisciplinary collaborations that are increasingly important in addressing global challenges. To this end, this study aims to examine research conducted on *maqāṣid al-shariī'ah* in journals indexed in Web of Science (WoS) and Scopus to determine the trending issues, hotspot areas, document co-citation analysis, active geographical regions, institutions, and publishing journals among others.

*Maqāṣid al-shariī'ah* is a significant field within Islamic *fiqh* (jurisprudence) and Islamic philosophy. The Arabic term *maqāṣid* can be literally translated into English as “goal,” “objective,” or “purpose”. However, in the context of Islamic jurisprudence, *maqāṣid* is concerned with the higher objectives and the noble aims of *shariī‘ah* (Islamic law). In other words, *maqāṣid al-shariī'ah* aims to identify the underlying intentions and goals of Islamic law and to explore the broader moral and ethical principles that guide Islamic legal rulings (Auda, 2008a; Rifai, 2021). Auda (2008b) gives a comprehensive definition of *maqāṣid al-shariī'ah*, defining it as the branch of Islamic knowledge that aims to address fundamental “why” questions at various levels and dimensions within Islam. It states the underlying reasons for Islamic obligations and practices, including the “why” of giving Zakah (obligatory almsgiving) and charity, the reasons behind performing the five daily prayers, and fasting during the month of Ramadan. *Maqāṣid al-shariī'ah* also aims to determine the objectives behind prohibiting alcohol and drugs, and imposing the death penalty for crimes such as intentional murder, rape, or genocide (Auda, 2008a). Additionally, *maqāṣid al-shariī'ah* explores the “wisdom behind rulings,” providing insights into the noble aims and objectives of Islamic laws. For instance, it explains how ṣ*adaqah* (voluntary charity), kindness to neighbors, and generosity and greeting with peace may enhance social cohesion. Moreover, *maqāṣid al-shariī'ah* explains that obligatory prayers, fasting during the month of Ramadan, voluntary fasting, and other supererogatory acts of worship aimed at developing consciousness of God (Auda, 2008a).

The traditional *maqāṣid al-shariī'ah* approach includes three tiers of essentiality: *daruriyat* (necessities), *hajiyyat* (needs), and *tahsiniyyat* (luxuries) (Auda, 2008a). Necessities are further subdivided into the preservation of *din* (religion), *nafs* (psyche, soul, etc.), *aql* (intellect), *nasl* (progeny), and *mal* (property) (Auda, 2008a, p. 3). Some scholars have added “the preservation of honour” to the above five necessities (Auda, 2008a, p. 3). *Maqāṣid al-shariī'ah* therefore adopts an interdisciplinary approach that draws on various Islamic sciences, including *aqidah* (theology), *fiqh* (jurisprudence), *ilm al-kalam* (philosophy), and *akhlaq* (ethics) (‘Athiyah, 2001). Additionally, *maqāṣid al-shariī'ah* studies incorporate insights from contemporary social sciences and ethics (Laldin, 2020).

The concept of *maqāṣid* has facilitated the formulation of contemporary, forward-thinking, and logical interpretations of Islamic law. By focusing on the higher objectives and aims of *shariī‘ah*, this approach aims to apply Islamic principles to the modern context and address issues and challenges that Muslims encounter today. As it allows for a more nuanced understanding of Islam, the *maqāṣid* approach is widely used in addressing contemporary challenges in Muslim communities as well as global contemporary problems. The application of *maqāṣid al-shariī'ah* is a clear indication of the dynamism of Islamic law and its continued relevance to the concerns of society.

It can be argued that *maqāṣid al-shariī'ah* is oriented toward the moderation of Islam. In situations where there may be conflicting rulings, the *maqāṣid* framework prioritizes higher objectives over specific legal details. Nevertheless, the *maqāṣid* framework has not been exempt from critique. Critics argue that the concept of *maqāṣid* is too subjective and open to interpretation, leading to potential misuse or manipulation. Moreover, *maqāṣid* jurists have been accused of bestowing legitimacy on political power; they use the *maqāṣid* approach to justify certain policies in the name of preserving the public interest (al-Marz ūqī, 2017). It displays arbitrary decisions with a clear lack of a vigorous method and transparent authority (Belhaj, 2023). Some conservative religious scholars reject the application of *maqāṣid* altogether, considering it to be a departure from strict adherence to traditional Islamic teachings. They argue that relying on *maqāṣid* can lead to compromising the integrity and authenticity of Islamic principles (Adnan, 2021).

A considerable number of studies have adopted the *maqāṣid* framework to address modern ethical and legal dilemmas, including social justice, human rights, ethics, politics, economics, health, and family, among others (Aminuddin Shofi et al., 2022). One of the fields to which *maqāṣid* discourse has been applied is the field of finance and investment. In this context, proponents of *maqāṣid* argue that applying its principles related to finance and investments can help ensure ethical practices and promote economic stability. In this view, by aligning financial activities with the higher objectives of justice, fairness, and social welfare, *maqāṣid* can contribute to a more sustainable and inclusive financial system (Al-Mubarak, 2016; Muhamad et al., 2022; Noh, 2022; Rosman et al., 2022; Satyakti, 2023).

*Maqāṣid al-shariī'ah* has also been widely used in medical discourses to address ethical considerations in healthcare and medical decision-making. Advocates of *maqāṣid al-shariī'ah* in the medical field argue that applying its principles can help prioritize patient wellbeing, ensure equitable access to healthcare services, and promote ethical conduct among healthcare professionals. By aligning medical practices with the higher objectives of compassion, justice, and preservation of life, *maqāṣid* can contribute to a more holistic and patient-centered approach to healthcare (Ghalia et al., 2018; Hashi, 2019; Alfahmi Manal, 2022).

*Maqāṣid al-shariī'ah* principles have also been used in political decision-making and governance (Rane, 2011b; Abdelgafar, 2018; Safiyanu Duguri et al., 2021). Studies in this area emphasize the importance of promoting social justice, upholding human rights, and ensuring the welfare of society as a whole. By incorporating a *maqāṣid* approach into political systems, policymakers can strive for policies that prioritize the common good and address the needs and aspirations of their citizens. This approach can lead to more inclusive and equitable societies.

Moreover, *maqāṣid al-shariī'ah* has been widely used in the discourse of *wasaṭiyyah* (middle way, or avoiding extremes) to highlight the significance of moderation and balance in political decision-making (Mohamed, 2018). It encourages policymakers to avoid extremism and promote harmony among diverse groups within society (Ali and Rafeeque, 2018). By adopting a *wasaṭiyyah* approach, political systems can foster tolerance, respect, and cooperation, ultimately contributing to the stability and prosperity of the nation.

Many studies have also applied *maqāṣid al-shariī'ah* to tackle family problems (Husni et al., 2015; Rahman and Mohammad Monawer, 2020; Hamid, 2021; Sedayu, 2022). *Maqāṣid al-shariī'ah* can play a significant role in guiding family, romance, and inheritance matters. By focusing on the principles of justice, compassion, and equality, *maqāṣid* may ensure that these aspects of life are approached in a fair and inclusive manner. This can contribute to stronger familial bonds, healthier relationships, and a more equitable distribution of wealth within communities.

The use of *maqāṣid al-shariī'ah* in initiatives to establish what some scholars have described as a “new Islamic discourse” can also promote a more progressive and inclusive understanding of Islam (Rishard et al., 2018; Yunus and Yusoff, 2021; Mujib and Hamim, 2022). By emphasizing the values of tolerance, respect, and social justice, these initiatives aim to challenge traditional interpretations that may perpetuate discrimination or marginalization. This can lead to a more harmonious coexistence among diverse communities and foster a sense of unity and solidarity among Muslims worldwide. The *maqāṣid* framework has also been employed in the creation and harmonization of positive contemporary laws (Kamali, 2007; Hedayati-Kakhki and Bohlander, 2009; Kazemi-Moussavi, 2010). Apart from the applications of *maqāṣid* in various fields, many studies have delved into the critical analysis of some lexical aspects of the field such as *sabab, maqṣid*, ‘*illah. ḥikmah*, ḥ*aqq*, and *ghayb* (Atiyah, 2007; Auda, 2008a; Kamali, 2008).

## 2 Methodology

### 2.1 Databases

Elsevier Scopus and Clarivate Web of Science (WoS) constitute the main sources for the data in this scientometric study. While many scientometric studies either use Clarivate Web of Science (WoS) or Scopus for the collection of data, this study opts for the use of both databases. It is the contention of the study that these different databases have unique strengths and specializations. For instance, Scopus may cover more journals in the fields of social sciences and humanities, while Web of Science (WoS) might have a stronger focus on natural and medical sciences. By using multiple databases, we ensure a wider and more inclusive coverage of the discipline of *maqāṣid al-shariī'ah* and its various applications.

Furthermore, a scientometric study on a discipline that spans across multiple databases like Scopus and Web of Science (WoS) is generally more robust and comprehensive than one confined to a single database. This approach leads to a more accurate and holistic understanding of the research landscape in a given discipline. In addition, accessing multiple sources increases the total volume of data, which can provide a more detailed picture of the research landscape, especially in a discipline like *maqāṣid al-shariī'ah* where studies in English are relatively few. Another reason for selecting these databases is that a significant number of publications on *maqāṣid al-shariī'ah* are found in Web of Science (WoS), particularly between 2000 and 2010, whereas Scopus shows fewer records of publications on this topic during this period. This indicates that Web of Science (WoS) has been a more consistent and reliable source for *maqāṣid*-related publications over a broader time range. The fact that the two databases allow downloading data in various forms and formats adds additional impetus for the selection. This data accessibility facilitates the use of various scientometric and bibliometric software.

This study is limited to original research articles on *maqāṣid al-shariī'ah* that were published between 2000 and 2022 in English. Review articles, conference papers, and book chapters were excluded using each platform's filtering options. Using simple search terms like *maqāṣid al-shariī'ah* with its various spellings helped to narrow down the scope of the chosen studies. It is worthwhile to mention that the various forms of the collocation *maqāṣid al-shariī'ah* identified in the data and used later in our analysis are not duplicates but rather distinct transliterations and diacritical variants of the original Arabic 
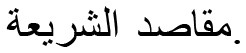
. These differences arise from the transliteration of Arabic script into the Latin alphabet, which can vary based on regional, linguistic or phonological conventions and the inclusion or exclusion of diacritical marks. For instance, terms such as “*maqasid al-shariah,” “maqāsid al-shari'ah,” “maqasid al-shari'ah,”* and “*maqasid al-shari'ah”* among others reflect these orthographic variations. Despite formal differences, all these terms/expressions refer to the same underlying Islamic legal concept but are treated as separate keywords in this analysis due to their distinct appearances in the scholarly literature.

### 2.2 Pre-processing brief and software

The following procedures have been followed in the pre-processing of data:

1. Publish or Perish (Harzing, 2007) has been initially used to source out research on *maqāṣid al-shariī'ah*. It is a widely used software in bibliometric analysis, especially in the retrieval and analysis of citation data from various sources, including Web of Science (WoS), Scopus, Google Scholar and Google Scholar profiles.
2. The datasets retrieved have been pre-processed by ScientoPy (Ruiz-Rosero et al., 2019), an open-source Python-based scientometric analysis tool. Some of the features of the tool include importing Clarivate Web of Science (WoS) and Scopus data, merging data sets from various databases, locating and removing duplicated documents, and presenting bibliometric data in different visualization graphs. The preprocessing brief is given in Table 1.
3. The preprocessing phase shows that two papers were identified as omitted from Scopus and 55 papers were identified as duplicates. However, since ScientoPy or similar reference managers are likely to identify duplicates based on multiple metadata fields, including, journal titles, DOI (Digital Object Identifier), author names, publication dates, we had to conduct separate searches in Scopus and Web of Science (WoS) directly as they allow more filtering options than third-party software. The following search query syntax was used to locate the publications and improve the accuracy and relevance of results in Scopus.

TITLE-ABS-KEY ( maqasid ) AND ( LIMIT-TO ( EXACTKEYWORD , “Maqasid Al-Shari'ah”) OR LIMIT-TO ( EXACTKEYWORD , “Maqasid Al-Shariah”) OR LIMIT-TO ( EXACTKEYWORD , “Maqasid”) OR LIMIT-TO ( EXACTKEYWORD , “Maqasid Al-shari'ah”) OR LIMIT-TO ( EXACTKEYWORD , “Maqasid Shariah”) OR LIMIT-TO ( EXACTKEYWORD , “Maqasid Shari'ah”) OR LIMIT-TO ( EXACTKEYWORD , “Maqasid Al-shariah”) OR LIMIT-TO ( EXACTKEYWORD , “Maslahah”) OR LIMIT-TO ( EXACTKEYWORD , “Maqasid Al-Shari'ah”) OR LIMIT-TO ( EXACTKEYWORD , “Maqasid Syariah”) OR LIMIT-TO ( EXACTKEYWORD , “Maqasid Al Shariah”) OR LIMIT-TO ( EXACTKEYWORD , “Maqasid Al-shari'a”) OR LIMIT-TO ( EXACTKEYWORD , “Maqasid Sharia”) OR LIMIT-TO ( EXACTKEYWORD , “Maqasid Al-Sharia”) OR LIMIT-TO ( EXACTKEYWORD , “Maqasid Al-Shari'a”) OR LIMIT-TO ( EXACTKEYWORD , “Maqasid Al Shari'ah”) OR LIMIT-TO ( EXACTKEYWORD , “Maqasid Al-syariah”) OR LIMIT-TO ( EXACTKEYWORD , “Maqasid Al-sharia”) OR LIMIT-TO ( EXACTKEYWORD , “Maqasid Al-Syariah”) OR LIMIT-TO ( EXACTKEYWORD , “Maqasid Al Sharia”) ) AND ( EXCLUDE ( LANGUAGE , “Arabic”) OR EXCLUDE ( LANGUAGE , “Indonesian”) OR EXCLUDE ( LANGUAGE , “Malay”) OR EXCLUDE ( LANGUAGE , “Russian”) OR EXCLUDE ( LANGUAGE , “Spanish”) OR EXCLUDE ( LANGUAGE , “Turkish”) ) AND ( EXCLUDE ( SRCTYPE , “b”) OR EXCLUDE ( SRCTYPE , “k”) OR EXCLUDE ( SRCTYPE , “p”)) AND ( EXCLUDE ( PUBYEAR , 2023 )).

**Table 1:** Pre-processing brief of data in ScientoPy.

^*****^Original data ^*****^		
Loaded papers	400	
Omitted papers by document type	2	0.5%
Total papers after omitted papers removed	398	
Loaded papers from WoS	205	51.5%
Loaded papers from Scopus	193	48.5%
„,WoS,”0, 0.0%”,”205, 51.5%”,”0, 0.0%”,”0, 0.0%”,”0, 0.0%”,”205, 51.5%”		
„,Scopus,”0, 0.0%”,”166, 41.7%”,”20, 5.0%”,”0, 0.0%”,”0, 0.0%”,”186, 46.7%”		
Duplicated removal results:		
Duplicated papers found	55	13.8%
Removed duplicated papers from WoS	1	0.5%
Removed duplicated papers from Scopus	54	28.0%
Duplicated documents with different cited by	44	80.0%
Total papers after rem. dupl.	343	
Papers from WoS	204	59.5%
Papers from Scopus	139	40.5%
Statics after duplication removal filter		
„,WoS,”0, 0.0%”,”204, 59.5%”,”0, 0.0%”,”0, 0.0%”,”0, 0.0%”,”204, 59.5%”		
„,Scopus,”0, 0.0%”,”114, 33.2%”,”18, 5.2%”,”0, 0.0%”,”0, 0.0%”,”132, 38.5%”		

That is to say, the query searches for documents where the term “maqasid” appears in the title, abstract, or keywords. It focuses on keyword variations of “Maqasid Al-Shari'ah” and related spellings, including “Maqasid,” “Maqasid Syariah,” and similar terms. The search query syntax excludes documents written in Arabic, Indonesian, Malay, Russian, Spanish, and Turkish languages. It filters out certain types of documents (books, conference papers, and proceedings). It excludes documents published in the year 2023.

To locate and retrieve publications from the Web of Science (WoS), the following search query was used.

maqasid (topic) and Maqasid Al-shari Ah (should – search within topic) and Maqasid Al-shariah (should – search within topic) and Maqasid Shariah (should – search within topic) and Maqasid Al-sharia (should – search within topic) and review article (exclude – document types) and open publisher-invited reviews and 2023 (exclude – publication years) and proceeding paper or book chapters or editorial material or book review or book or early access (exclude – document types).

This search query syntax looks for documents on the topic “Maqasid” and related variations such as “Maqasid Al-Shari'ah,” “Maqasid Shariah,” and similar terms. It excludes review articles, publisher-invited reviews, and documents published in 2023. It also filters out proceeding papers, book chapters, editorial materials, book reviews, books, and early access documents.

4. The two datasets have been manually verified. Even though the search query excluded publications from 2023, the screening process shows 7 publications in Web of Science (WoS). These publications might have appeared in the dataset with a publication year of 2023 due to a common practice in academic publishing known as early online publication or pre-publication release.

In short, this scientometric study is limited to English journal articles that were published between 2000 and 2022 on *maqasid* research in Scopus and Web of Science (WoS). Given the dynamic nature of these two databases, where results can fluctuate rapidly due to the continuous addition of new papers or the retraction of some existing articles, this study is limited to the datasets that were created at the time of investigation. The investigation was finally resolved on Sunday, 03 December 2023, 16:07:52, yielding 205 documents from the Web of Science (WoS) and 195 documents from Scopus, as shown in Table 2.

5. VOSviewer (Van Eck and Waltman, 2017) has been used in our sientometric analysis. It is specifically useful in the visualization of data and bibliometric networks. It enables the identification of key themes, citations, sources, publishers, authors, and collaborations within the field of *maqāṣid al-shariī'ah*.

**Table 2:** Number of publications in the two databases.

**Year**	**Scopus publications**	**WoS publications**
2000	0	0
2001	0	0
2002	0	0
2003	0	0
2004	0	0
2005	0	0
2006	0	0
2007	0	0
2008	0	1
2009	0	1
2010	2	2
2011	3	2
2012	4	0
2013	6	1
2014	10	5
2015	14	10
2016	9	7
2017	19	3
2018	26	44
2019	22	28
2020	25	24
2021	29	37
2022	26	33
2023	0	7
Total	195	205

## 3 Results

**Figure 1:**
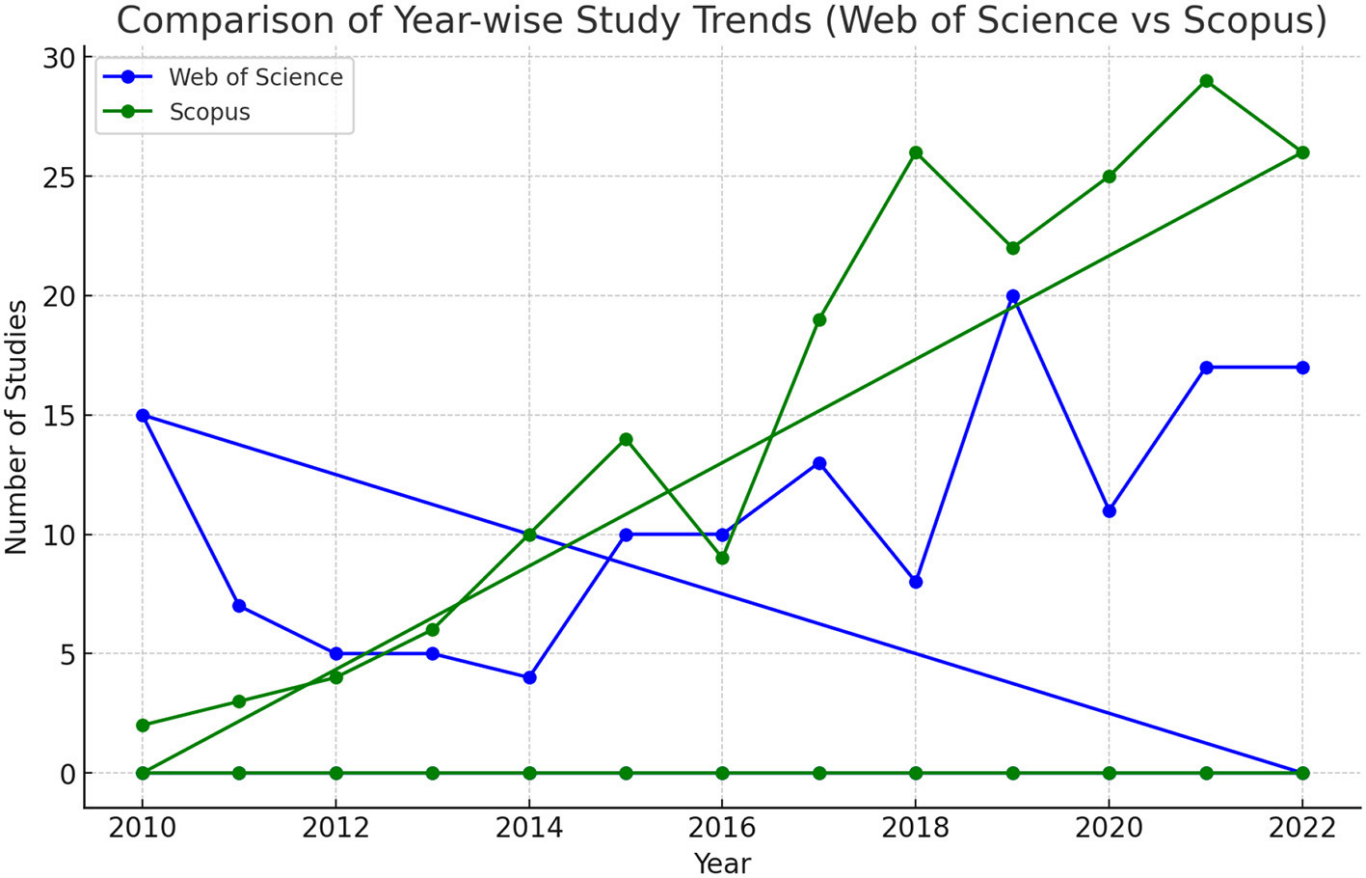
Publication performance in the two databases.

International scholarly literature in *maqāṣid al-shariī'ah* has been steadily increasing in recent years on a global scale. The comparison of year-wise study trends between the Web of Science (WoS) and Scopus datasets, is shown in Figure 1.

As Figure 1 shows, the development of publications in both Scopus and Web of Science (WoS) databases indicates distinct upward trends in cumulative publications and annual growth. The plot shows how both datasets have seen an increasing number of studies over the years, with similar peaks around 2019–2021. Scopus appears to have a more rapid rise in publications starting in 2017, while Web of Science (WoS) shows a consistent increase starting earlier.

Research outputs in *maqāṣid al-shariī'ah* span over various disciplines including religion, business and economics, science and technology, and medicine, among others. Table 3 shows the top 10 disciplines in which the *maqāṣid* framework is applied.

**Table 3:** Top 10 disciplines which include *Maqāṣid al-shariī'ah*.

**Subject**	**Total publications**
Religion	79
Business & Economics	71
Social Sciences - Other Topics	24
Science & Technology - Other Topics	14
Government & Law	12
History & Philosophy of Science	11
Philosophy	10
Engineering	9
General & Internal Medicine	8
Medical Ethics	7

### 3.1 Co-keyword and keyword citation bursts analysis

To illustrate the research hotspots in *maqāṣid* studies, keyword co-occurrence was analyzed with VOSviewer. The threshold was set as one document. It was noticed that a visualization may become too sparse or limited, should thresholds be too high. We have therefore opted for lowering the thresholds to figure out more detailed and complex networks, which is beneficial for a better understanding of the research landscape in *maqāṣid* studies.

**Figure 2:**
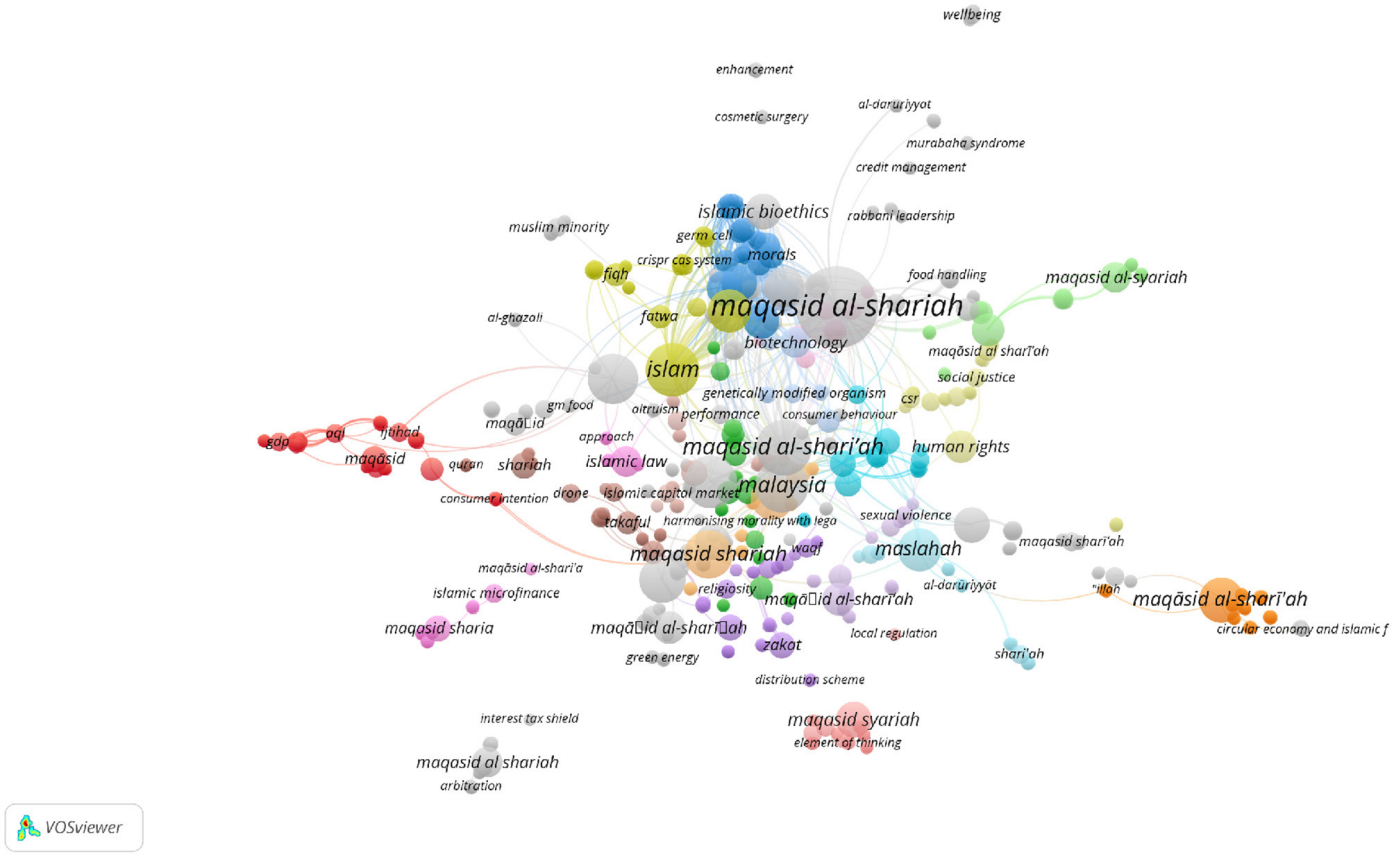
Occurrences-based co-keyword network visualization (Scopus).

**Figure 3:**
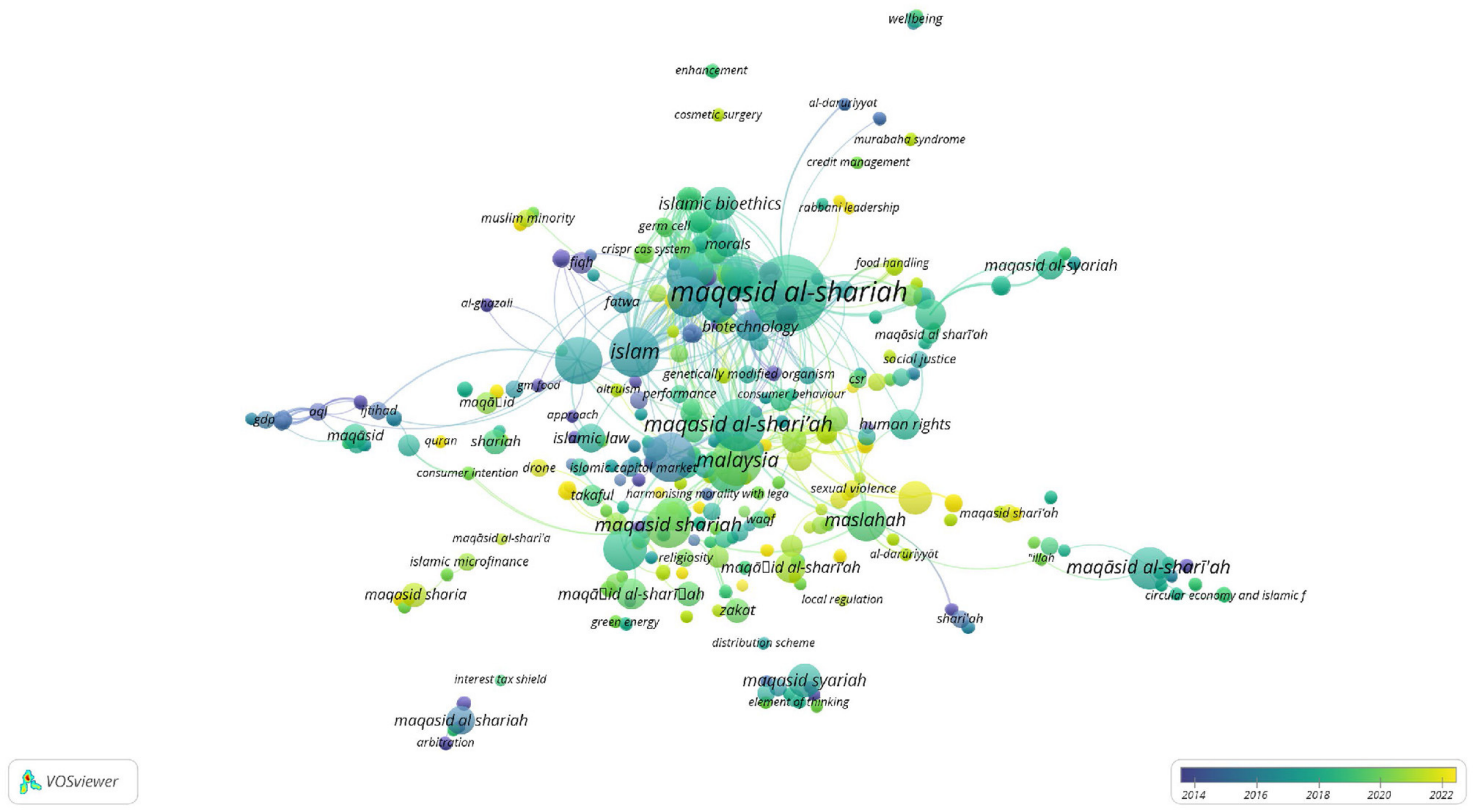
Co-keyword overlay visualization in Scopus (occurrences and average annual publication scores).

In Scopus, the keywords in total were 781 as shown in the co-keyword network visualization in Figure 2 and the co-keyword overlay visualization in Figure 3. The former is based on occurrences and the latter is based on the occurrences and average annual publication scores.

The link and total link strength information of the top 20 occurrence keywords are given in Table 4.

**Table 4:** The link and total link strength of the top 20 occurrence keywords in Scopus.

**Keyword**	**Occurrences**	**Total link strength**
maqasid al-shariah	37	220
maqasid al-shari'ah	17	134
Malaysia	16	97
Islam	16	212
maqasid al-shari'ah	15	73
Maqasid	14	94
maqasid shariah	13	52
islamic finance	12	54
maqāsid al-shari'ah	11	45
Maslahah	10	56
islamic banking	10	55
Ethics	10	167
Human	9	176
Religion	7	135
maqasid Syariah	7	23
islamic bioethics	7	95
islamic banks	7	28
Humans	7	141
Covid-19	7	51
Bioethics	7	117

Figures 2, 3, and Table 4 provide an overview of the most frequent keywords pertaining to *maqāṣid al-shariī'ah*. They highlight the key themes and areas of interest in this field of Islamic theology. The keyword “maqasid al-shariah” tops the list with 37 occurrences and a substantial link strength of 220. This keyword/phrase, along with its variants “maqasid al-shari'ah” (17 occurrences, 134 link strength), “maqasid al-shari'ah” (15 occurrences, 73 link strength), “maqasid shariah” (13 occurrences, 52 link strength), and “maqāsid al-shari'ah” (11 occurrences, 45 link strength), dominate the research landscape. These scholarly studies focus on the core objectives and goals of *Shari‘ah*. They reflect ongoing debates and discussions within the Islamic scholarly community. The variations in spelling indicate a diverse but concentrated scholarly interest in *maqāṣid al-shariī'ah* and the critical role it plays in shaping Islamic thought.

The results also highlight that the keyword “Malaysia” stands out as an important geographical focus in maqāṣid-related research, with 16 occurrences and a significant link strength of 97. This demonstrates Malaysia's leadership and innovation in implementing *maqāṣid al-shariī'ah* principles, especially in sectors like Islamic finance and banking. This finding is supported by the presence of related keywords such as “Islamic finance” (12 occurrences, 54 link strength) and “Islamic banking” (10 occurrences, 55 link strength), which represent key sectors where *maqāṣid* principles are applied to ensure that financial practices align with Islamic ethics and laws.

The inclusion of “Islam” as a prominent keyword, with 16 occurrences and a link strength of 212, indicates a broader exploration of Islamic jurisprudence and moral philosophy in the context of *maqāṣid al-shariī'ah* studies. This suggests a comprehensive approach to understand how Islamic teachings influence various aspects of life and governance. The integration of Islamic legal principles with ethical considerations has become prevalent over the last two decades.

An interesting development is the appearance of “COVID-19” as a keyword, with 7 occurrences and a link strength of 51. This reflects the responsiveness of *maqāṣid al-shariī'ah* to current global challenges, such as the COVID-19 pandemic. The presence of this keyword suggests an exploration of how Islamic principles can inform responses to pandemic situations. An Islamic response to COVID-19 and other pandemics touch on issues of public health, social responsibility, and ethical governance.

Finally, the occurrence of keywords like “maslahah” (public interest) with 10 occurrences and 56 link strength, “human” with 9 occurrences and 176 link strength, and “Islamic bioethics” with 7 occurrences and 95 link strength suggests that *maqāṣid al-shariī'ah* extends beyond legal and financial contexts. These keywords highlight its relevance in broader ethical and philosophical discussions, particularly in areas that intersect with public interest, human wellbeing, and bioethics.

**Figure 4:**
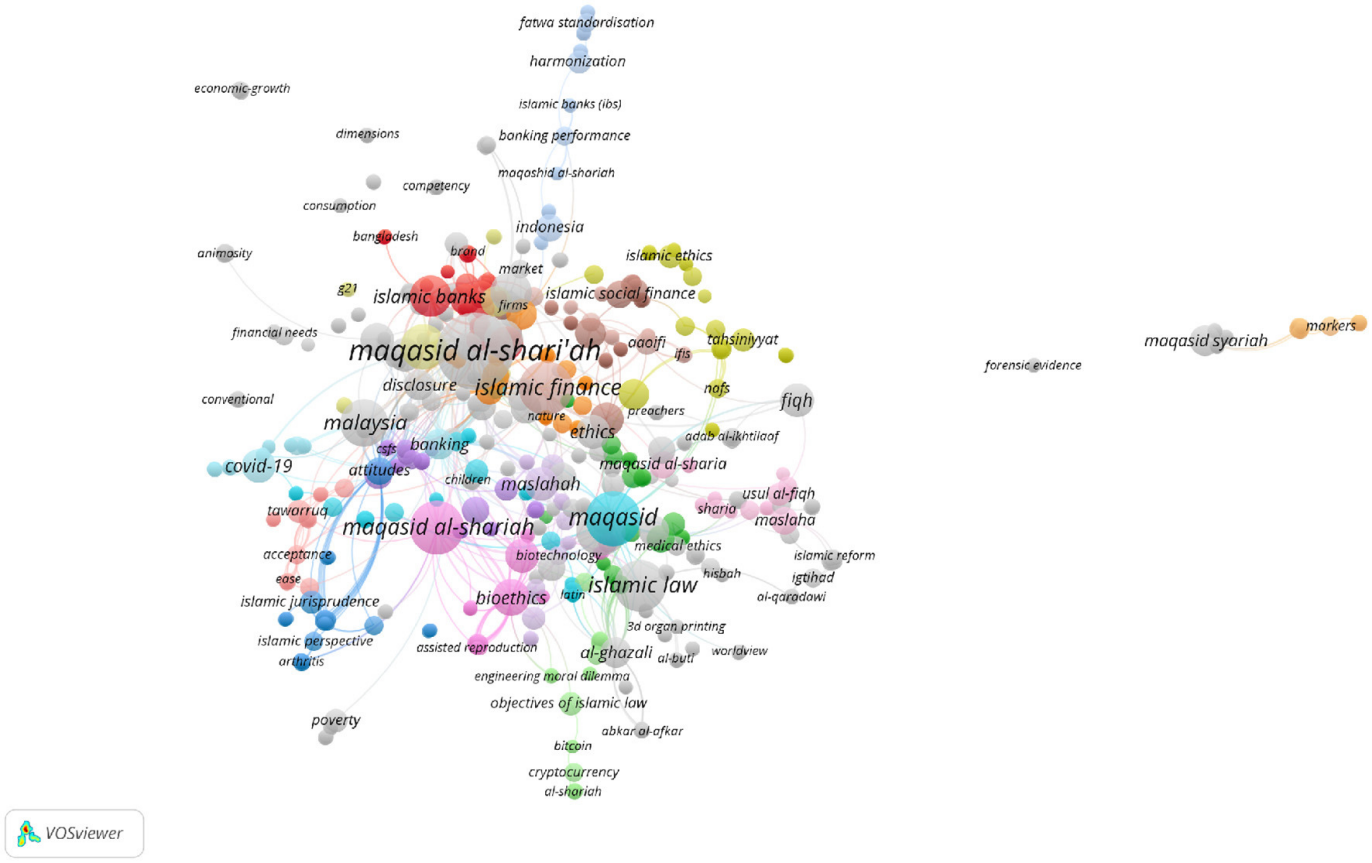
Occurrences-based co-keyword network visualization (WoS).

**Figure 5:**
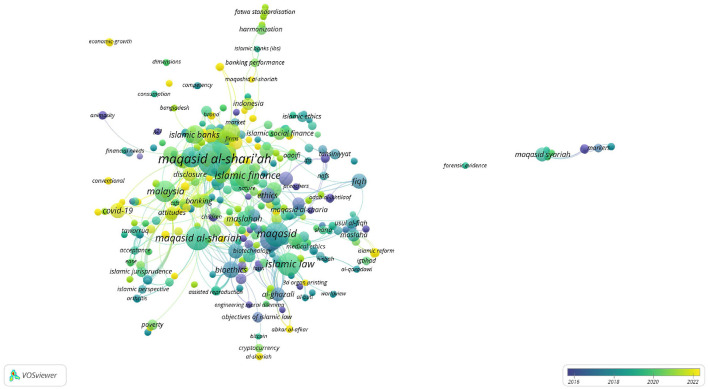
Co-keyword overlay visualization in WoS (occurrences and average publication per year scores).

Insofar as the landscape of *maqāṣid al-shariī'ah* studies in Web of Science (WoS) is concerned, the total keywords are 942, as shown in the co-keyword network visualizations in Figure 4 and the co-keyword overlay visualization shown in Figure 5.

The link and total link strength information of the top occurrence keywords in Web of Science (WoS) are given in Table 5.

**Table 5:** The link and total link strength of the top occurrence keywords in WoS.

**Keyword**	**Occurrences**	**Total link strength**
Maqasid al-shari'ah	31	168
Maqasid	16	118
Maqasid al-shariah	15	102
Islamic finance	15	100
Islamic law	14	63
Islam	13	81
Malaysia	12	72
Islamic banking	11	74
Performance	9	84
Islamic banks	9	61
Maqasid shariah	8	55
Bioethics	7	46
Religiosity	6	59
Maslahah	6	41
Islamic bioethics	6	45
Impact	6	72
Framework	6	42
Fiqh	6	37
Ethics	6	47
Determinants	6	58
COVID-19	6	26
Waqf	5	27
Shari'ah	5	43
Maqasid syariah	5	30
Islamic bank	5	37
Ijtihad	5	37
Governance	5	48
Financial performance	5	53
Finance	5	38
Disclosure	5	45
Banking	5	43
Al-ghazali	5	39

As is the case in the Scopus database, the term “maqasid al-shari'ah” emerges as the most frequently occurring keyword, with 31 occurrences, linked to 168 other terms. Closely related terms, “maqasid al-shariah” (15 occurrences, 102 link strength) and “maqasid” (16 occurrences, 118 link strength), also show significant occurrences and link strengths. The use of these keywords and expressions reflects a strong focus on the objectives of Islamic law. It highlights the central role *maqāṣid al-shariī'ah* plays in scholarly discussions on Islamic law and its broader implications.

“Islamic finance” and “Islamic banking” demonstrate the field's special emphasis on financial aspects, with 15 occurrences and 100 link strength for “Islamic finance” and 11 occurrences and 74 link strength for “Islamic banking”. These notable links and occurrences state the complexity and interdisciplinarity of the field, which integrates financial studies into broader discussions on Islamic law and ethics.

Further down the list, keywords such as “Islam” and “Islamic law” explain the foundational elements of *maqāṣid* studies. “Islam” appears with 13 occurrences and a link strength of 81, while “Islamic law” is noted with 14 occurrences and a link strength of 63. These keywords emphasize the importance of traditional Islamic teachings in the formation and application of *maqāṣid* principles.

In a similar trend observed in the Scopus database, “Malaysia” emerges as a strong geographical focus, with 12 occurrences and a link strength of 72. This indicates that Malaysia continues to be a significant center for *maqāṣid al-shariī'ah* studies, particularly in the fields of Islamic finance and banking.

Keywords such as “ethics” (6 occurrences, 47 link strength), “religiosity” (6 occurrences, 59 link strength), and “covid-19” (6 occurrences, 26 link strength) reflect the diverse range of research interests in the field of *maqāṣid al-shariī'ah*. These keywords highlight the field's engagement with moral principles, personal belief systems, and contemporary global challenges, such as the COVID-19 pandemic. This demonstrates the relevance of *maqāṣid* studies in addressing both traditional and modern issues.

Keywords such as “performance” (9 occurrences, 84 link strength), “governance” (5 occurrences, 48 link strength), and “impact” (6 occurrences, 72 link strength), especially when connected to “Islamic banks” (9 occurrences, 61 link strength) and “finance” (5 occurrences, 38 link strength), underscore the practical and evaluative aspects of Islamic finance and law. This indicates that *maqāṣid al-shariī'ah* studies are increasingly concerned with assessing the outcomes and efficiencies of Islamic banking systems, bridging theory with practice.

Finally, the occurrence of keywords such as “Islamic bioethics” (6 occurrences, 45 link strength) reflects the intersection of Islamic principles with modern ethical considerations in medicine and science. This suggests that *maqāṣid al-shariī'ah* offers a multifaceted approach that is applicable across various aspects of life, including contemporary bioethical dilemmas.

**Figure 6:**
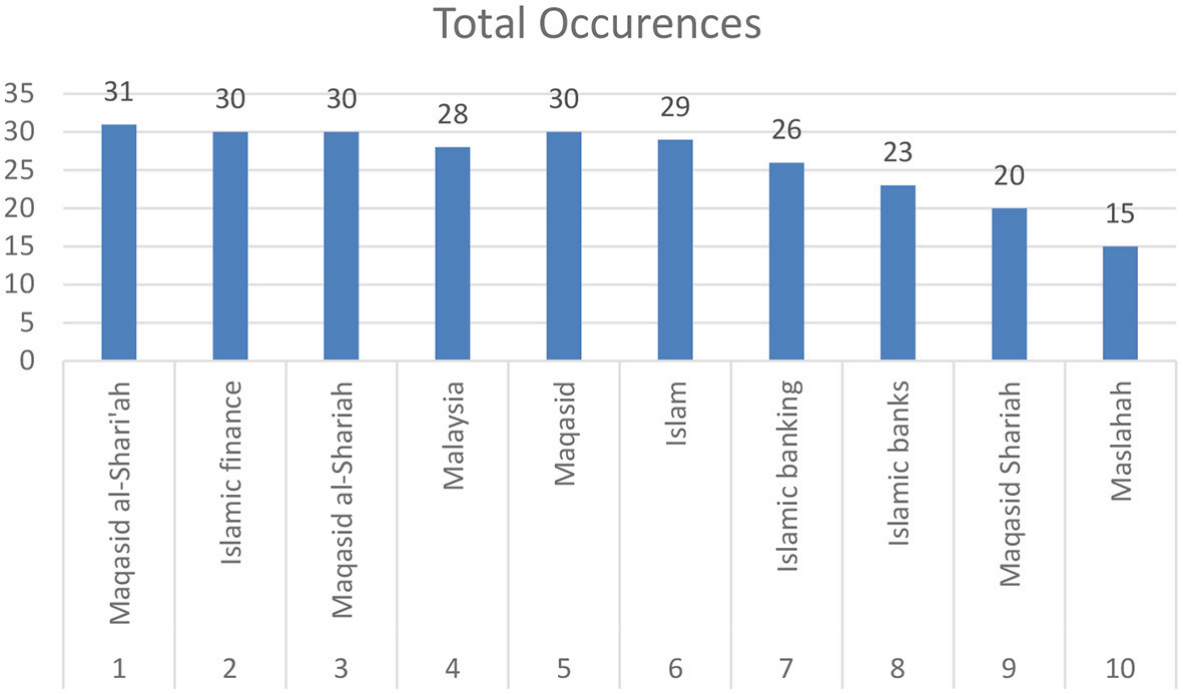
The top 10 keywords across the two databases.

Following a separate analysis of keywords and themes within each database, a comprehensive overview of the major keywords and themes prevalent across both databases is presented in Figure 6, which shows the top 10 keywords across the two databases.

#### 3.1.1 Word clouds

**Figure 7:**
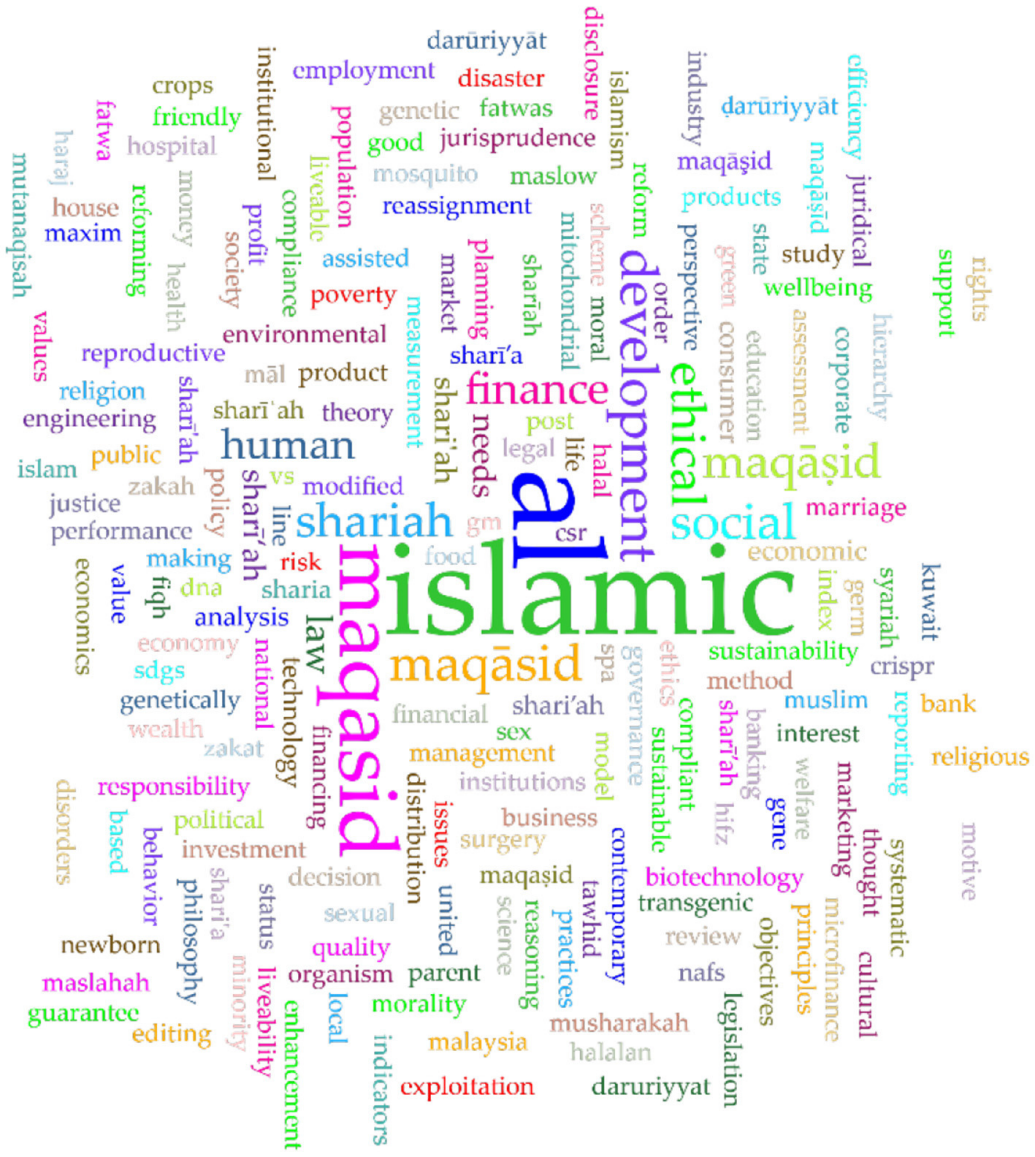
Word cloud of keywords in Scopus.

In addition to identifying the top keywords in both databases and across them, as discussed in previous sections, it is also beneficial to visualize these keywords using word clouds. Word clouds offer a graphical representation of the most frequent terms in a corpus or dataset, where the size of each word corresponds to its frequency (Bonkra et al., 2023, 2024; Mohammed, 2023). This method is often employed in scientometric and bibliometric studies to highlight key themes, trends, subjects, and research areas within a specific field. Word clouds provide a quick, visual summary of the content of large datasets, making complex information more digestible. Figure 7 presents the word cloud of the title and author's keywords in Scopus, as visualized by Voyant Tools.

**Figure 8:**
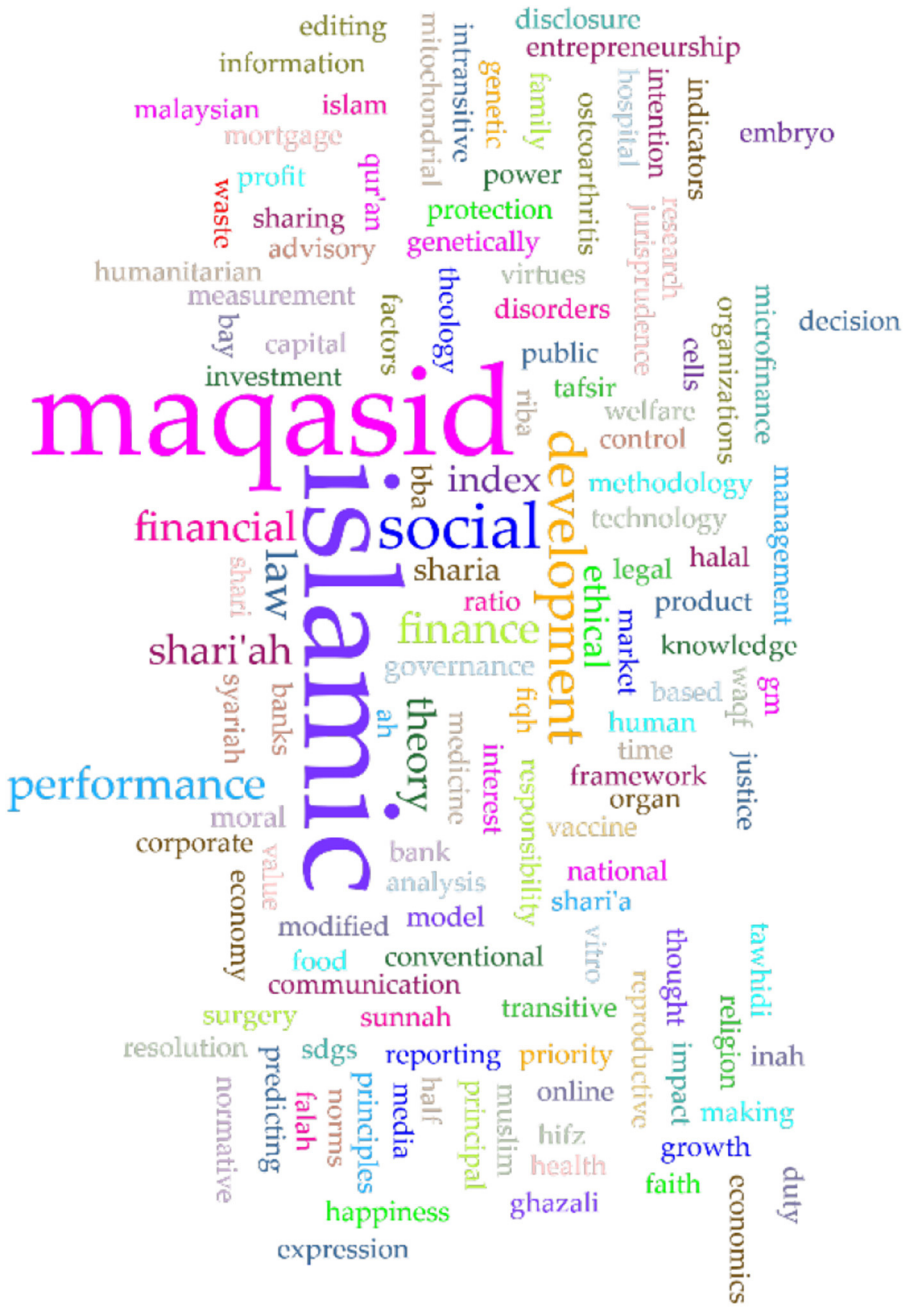
Word cloud of keywords in Web of Science.

In a similar vein, a word cloud of keywords in Web of Science (WoS) is given in Figure 8.

### 3.2 Co-authorship visualization analyses

**Figure 9:**
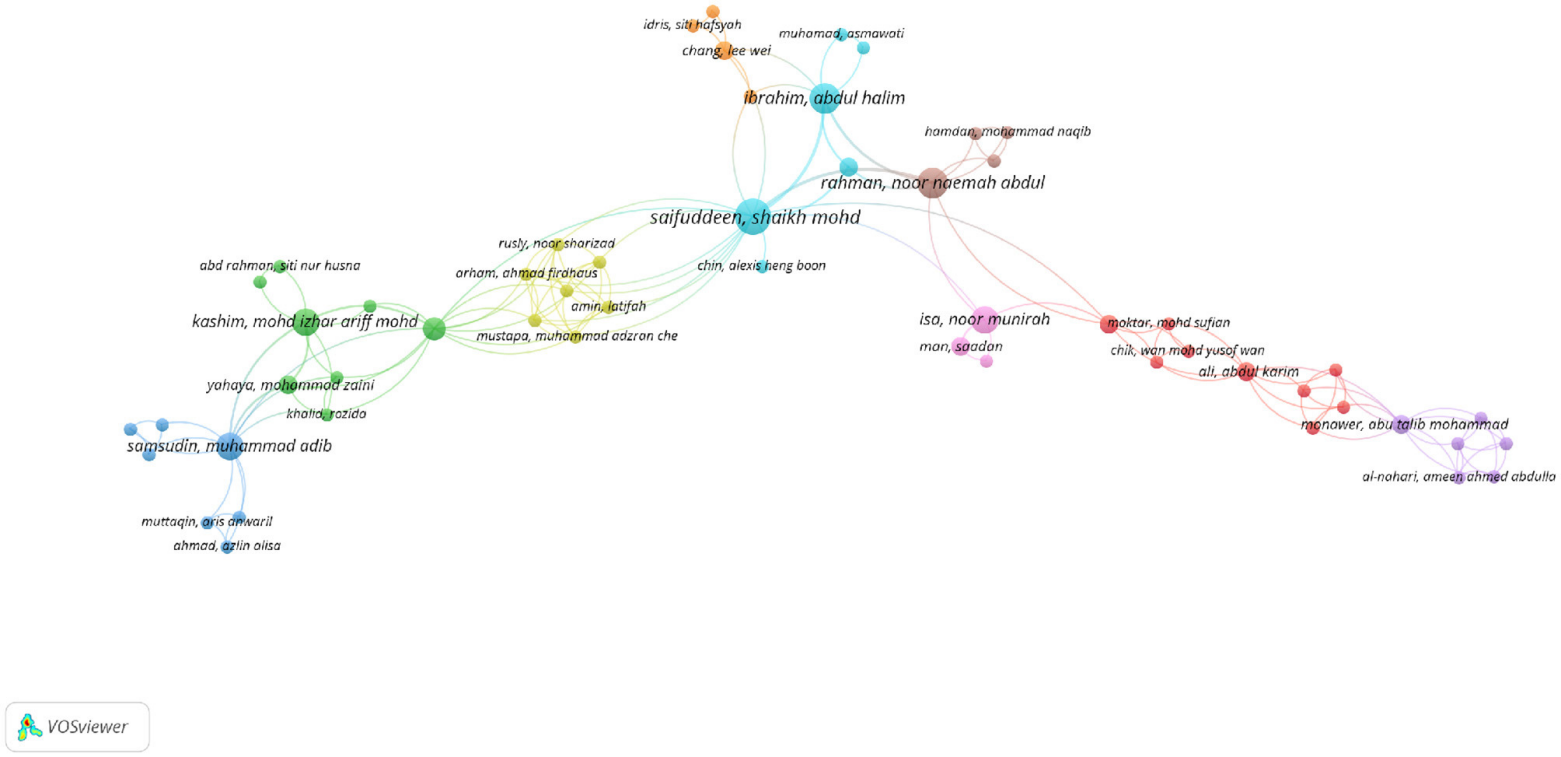
Authors network visualization in WoS.

Based on the co-authorship map shown in Figure 9, there were 419 authors in Web of Science (WoS).

In terms of total link strength, Saifuddeen, Shaikh Mohd (seven documents, 55 citations, and 23 total link strength) had the most influence, followed by Rahman, Noor Naemah Abdul (5 documents, 47 citations, and 14 total link strength) with the second most influence. Hassan, M. Kabir (5 documents, 150 citations, and 11 total link strength) had the third most influence.

**Figure 10:**
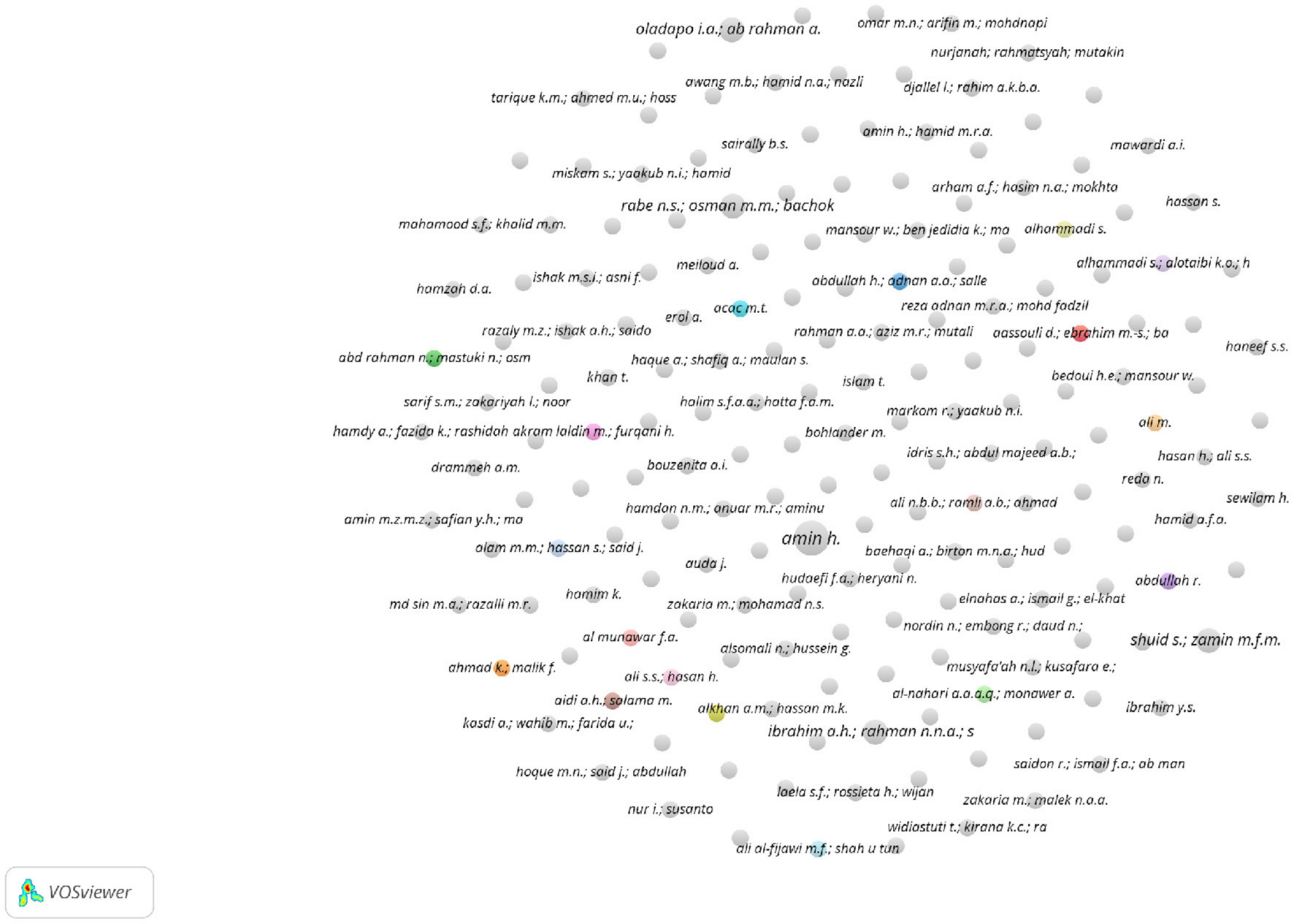
Authors network visualization in Scopus.

Exploring the co-citation scenario in Scopus shows that 188 authors meet the threshold of one document, as shown in the co-authorship map in Figure 10.

In terms of the total number of citations, “Akram Laldin M.” and “Furqani H.” top the list (68 citations), followed by “Alam M.M.” (29 citations), “Alkhan A.M.” and “Hassan M.K.” (18 citations). However, as the visualization indicates, there are no connections or relationships identified between the listed documents or authors. That is, the “total link strength” is 0 for each document. Typically, link strength in scientometric/bibliometric or academic databases refers to the degree of connection between documents, authors, or keywords, often based on citations, co-authorships, or thematic similarities. A link strength of 0 suggests that, despite the documents having individual citations, they have not been directly cited together, do not share authors, or have not been linked through common keywords in the database's analysis. This could be for several reasons including single-document authors, isolation of research topics, and new or emerging research areas. Furthermore, the field might still be in the early stages of development in terms of building a network of citations and connections.

**Figure 11:**
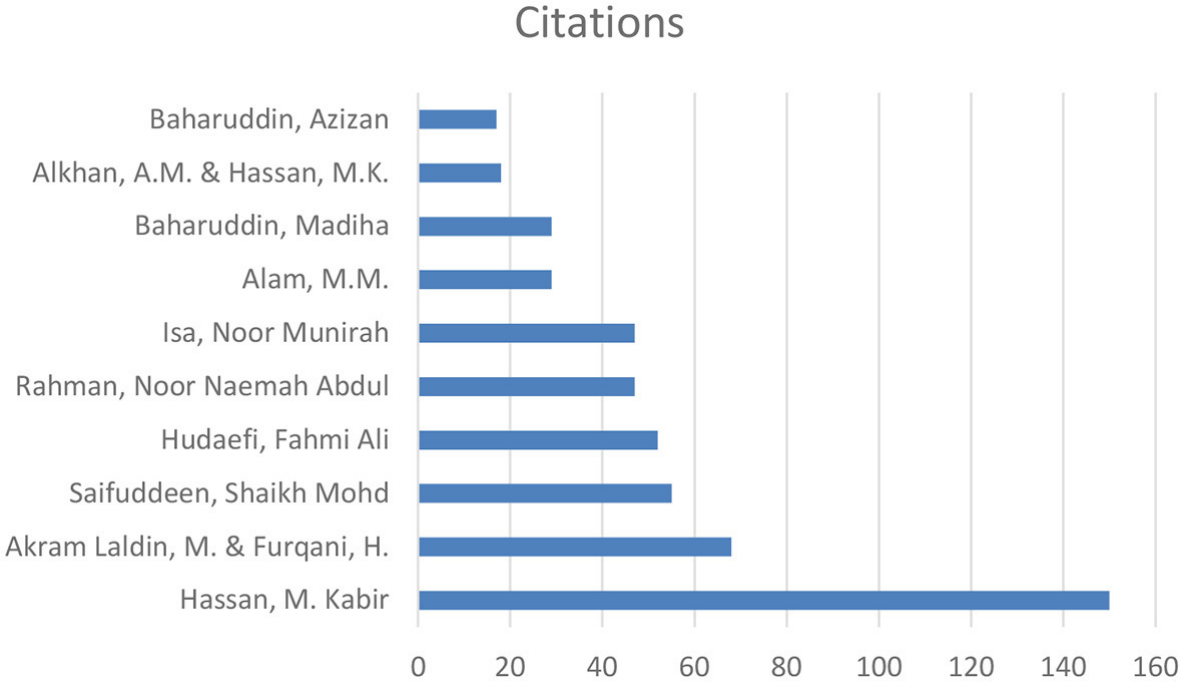
Top 10 authors across the two databases.

Figure 11 shows the top 10 authors across the two databases.

### 3.3 Co-author visualization map of countries/regions

**Figure 12:**
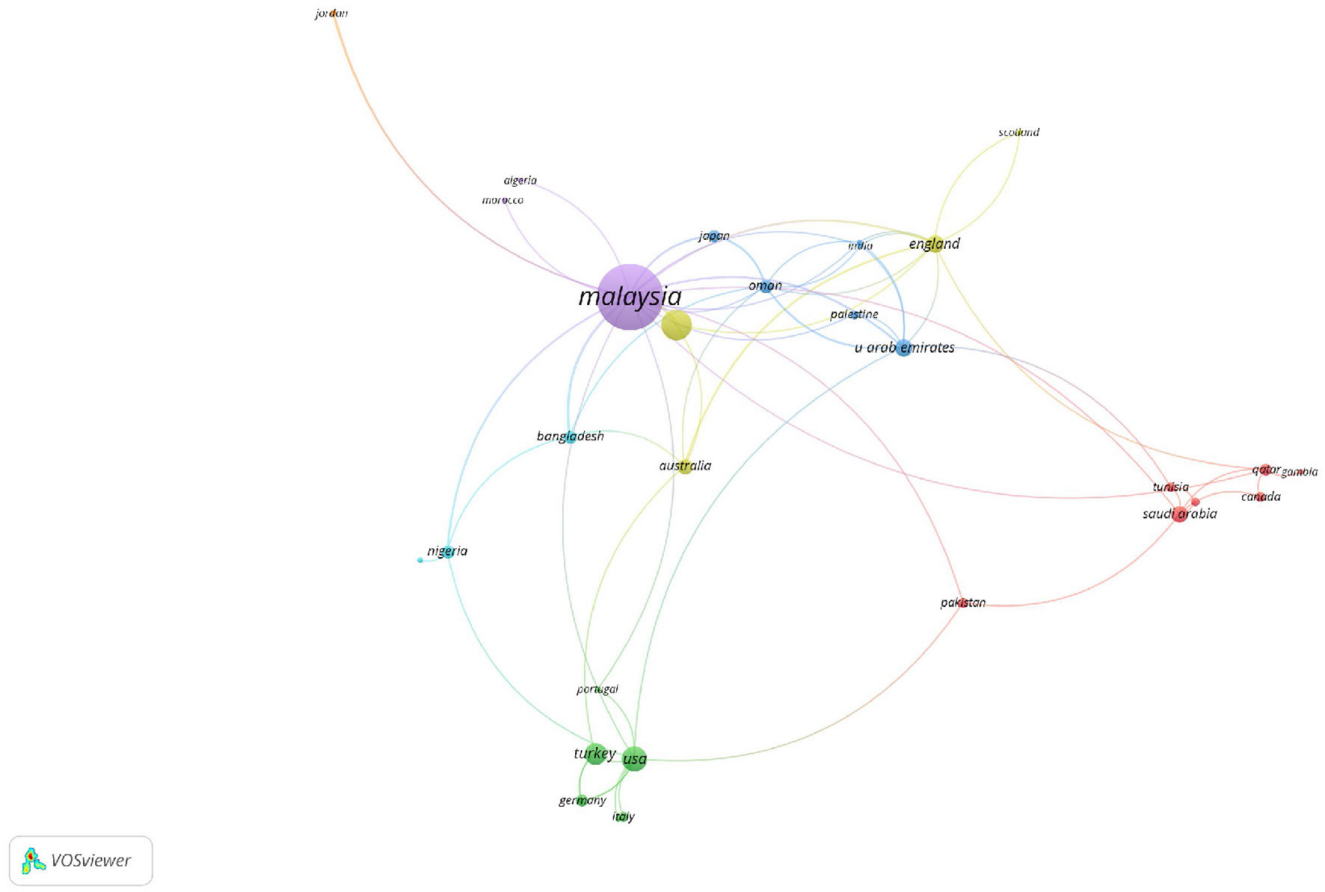
Co-author network visualization map of countries/regions in WoS.

In terms of the co-authorship map of countries in Web of Science (WoS) (Figure 12), it is important to note that 38 countries on the co-authorship map met the minimum threshold of at least one document for co-authorship.

As Figure 12 shows, Malaysia emerges as the leading country in *maqāṣid* studies by a substantial margin, evidenced by the highest number of documents (114) and citations (458), alongside a considerable total link strength of 30. This clearly highlights Malaysia's dominance and leadership in the field, particularly in sectors like Islamic finance and *maqāṣid*-related research. Indonesia follows as the second most prolific country, with 25 documents and 78 citations. This trend shows a vibrant scholarly interest in *maqāṣid* studies in Indonesia. Despite having fewer documents than Malaysia, Indonesia's contributions reflect a significant regional engagement with the subject. The United States, despite having fewer documents (17) compared to Malaysia and Indonesia, shows a high citation count (214). These figures indicate the influential nature of the research conducted there. That is, while the volume of *maqāṣid*-related research may be smaller, the impact of U.S. studies is substantial. England also demonstrates a notable contribution to the field, with 8 documents and 100 citations, alongside a strong total link strength of 12. These statistics indicate active scholarly participation and influence in *maqāṣid* research. Countries like Australia (6 documents, 25 citations), Bangladesh (5 documents, 29 citations), Germany (4 documents, 8 citations), and Oman (5 documents, 25 citations, and 10 total link strength) are examples of diverse contributors to *maqāṣid* studies. These countries represent growing interest in *maqāṣid al-shariī'ah* principles across a variety of global contexts, including both academic and practical applications.

The diversity of countries that contribute to *maqāṣid* studies from regions such as the Middle East (Saudi Arabia, United Arab Emirates, Oman), Asia (Malaysia, Indonesia, Turkey, Pakistan, Bangladesh), and Western nations (USA, England, Australia, Canada, Germany), highlights the global relevance of *maqāṣid al-shariī'ah* and its interdisciplinary applications. This geographical spread emphasizes the importance of *maqāṣid* studies in addressing both traditional Islamic legal principles and modern global challenges.

**Figure 13:**
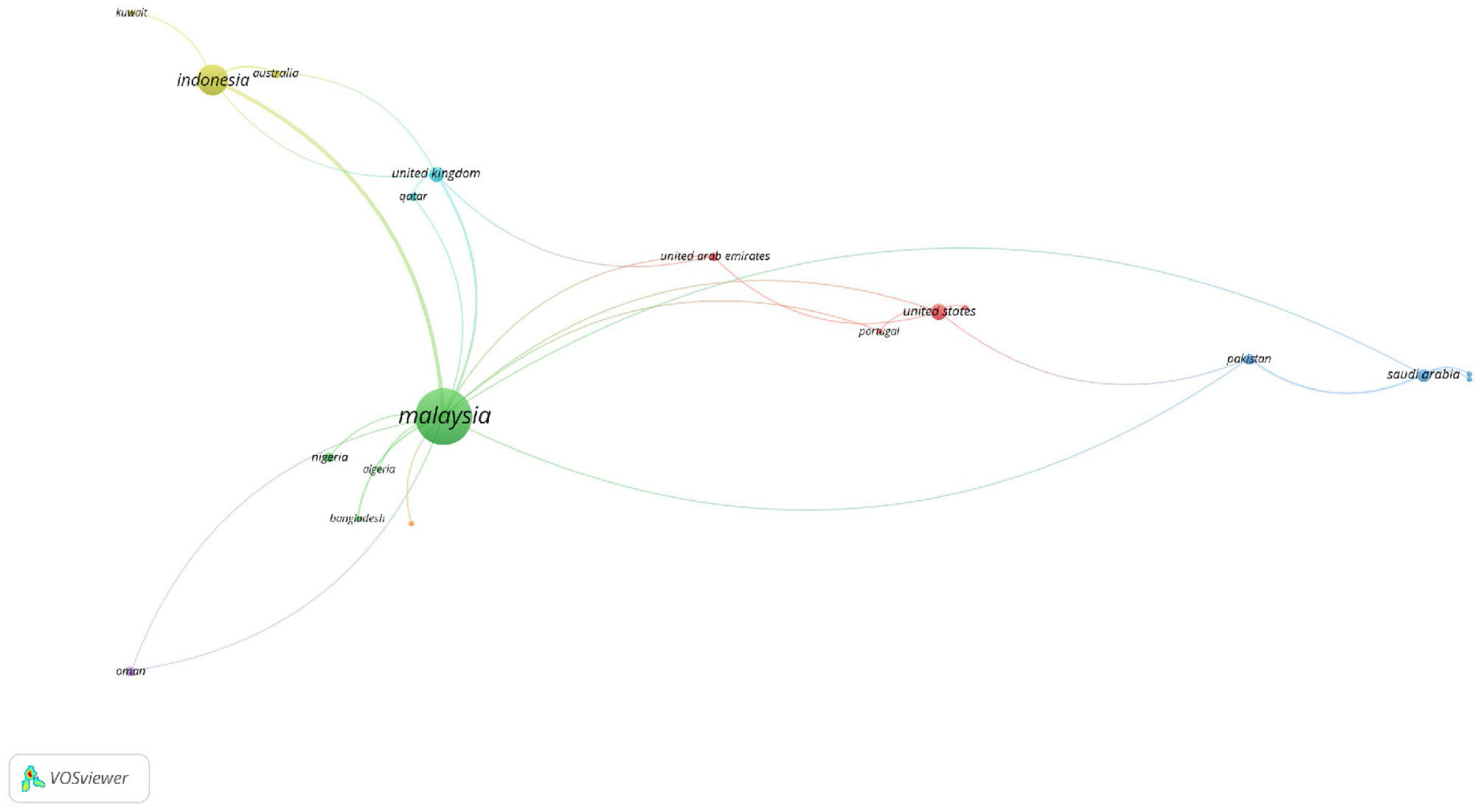
Co-author network visualization map of countries/regions in Scopus.

Scopus shows similar trends to a great extent as shown in Figure 13. 24 countries on the co-authorship map met the minimum threshold of 1 document for co-authorship.

As is the case in Web of Science (WoS), Figure 13 shows a vibrant and diverse global research landscape for *maqāṣid* studies, with Malaysia and Indonesia leading in terms of productivity and impact. Malaysia has the highest document count, with 123 documents and 742 citations, alongside a total link strength of 24. Indonesia follows with 39 documents, 198 citations, and a total link strength of 12. The United Kingdom and the United States show moderate levels of activity with 9 and 10 documents, respectively. The UK has 62 citations and a total link strength of 7, while the US has 74 citations and a total link strength of 5. Although the document counts are not the highest, their contributions remain significant, reflecting their engagement in *maqāṣid* research. The UK's research output is recognized as impactful within the broader academic landscape. Moreover, Saudi Arabia, with 7 documents and 116 citations, demonstrates its significance in *maqāṣid* research. Similarly, countries like Pakistan (5 documents, 22 citations), Nigeria (4 documents, 5 citations), Oman (4 documents, 38 citations), and Qatar (3 documents, 46 citations) indicate the diverse and international interest in *maqāṣid* principles and their applications.

Emerging contributions from Australia (3 documents, 23 citations), the United Arab Emirates (3 documents, 7 citations), Brunei Darussalam (2 documents, 11 citations), Canada (2 documents, 6 citations), and France (1 document, 49 citations), despite their lower document counts, suggest a global spread and interest in *maqāṣid* studies beyond predominantly Muslim countries. As stated earlier, this diversity indicates the universal appeal and relevance of *maqāṣid* principles in addressing contemporary global issues.

### 3.4 Organizations

**Figure 14:**
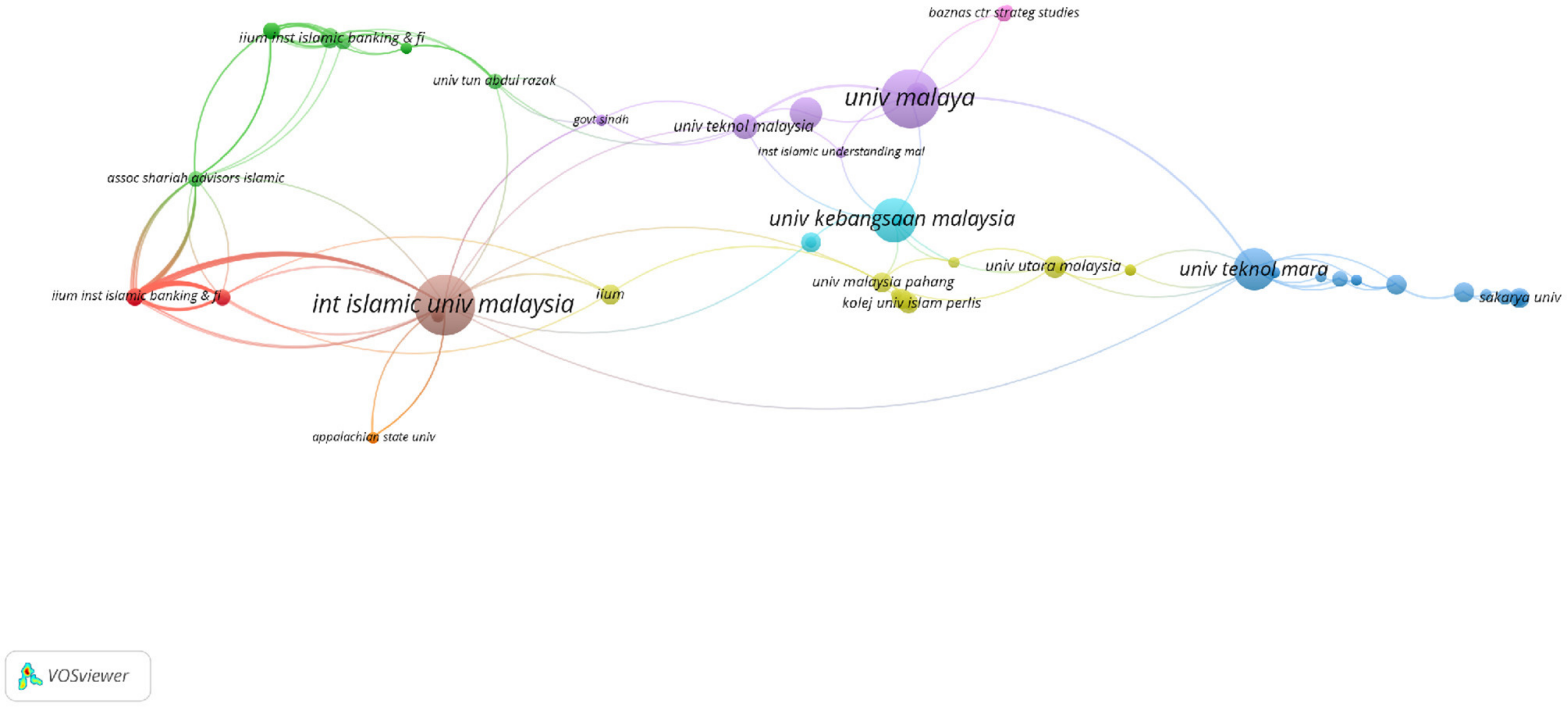
Co-author map of organizations in WoS.

A co-author map of organizations is essential for an analysis of the contributions and intellectual proximity of major research institutions in a particular discipline. In the context of *maqāṣid* studies in Web of Science (WoS), 230 organizations met the threshold of one document (Figure 14).

According to Figure 14, the keyword phrase “University of Malaya” (UM) has a notable position, with 26 documents, 138 citations, and a total link strength of 18. The International Islamic University Malaysia (IIUM) follows with 28 documents, 135 citations, and a total link strength of 43. UM and IIUM stand out as two of the most active institutions in *maqāṣid* studies, with significant contributions to the field.

Moreover, it can be observed that the most active cluster of research relationships revolves around several Malaysian and international institutions. These include “Institute of Islamic Understanding Malaysia (IKIM)”, “Universiti Teknologi MARA (UiTM)”, “Universiti Malaysia Sabah (UMS)”, “Universiti Kebangsaan Malaysia (UKM)”, “University of New Orleans”, “Universiti Teknologi Malaysia (UTM)”, “Durham University”, “Universiti Sains Islam Malaysia (USIM)”, “International Islamic University Malaysia (IIUM)”, “Universiti Sains Malaysia (USM)”, and “Islamic Development Bank (IsDB)”, among others. This group of institutions forms a dynamic network of knowledge generation in *maqāṣid* studies.

The University of Malaya leads with the highest number of citations (138 citations). This demonstrates its pivotal role in contributing to scholarly discussions in the field of *maqāṣid* studies. The International Islamic University Malaysia (IIUM) is close behind in both citations and total link strength, reflecting the institution's strong influence in this field of *maqāṣid* studies. Moreover, international institutions such as the University of New Orleans (5 documents, 150 citations) and Durham University (three documents, 50 citations) highlight the global spread of *maqāṣid* studies.

In general, while many international organizations are actively involved in the generation of knowledge in *maqāṣid* studies, Malaysian organizations show a clear dominance. This underscores Malaysia's leadership in the development and application of *maqāṣid* principles, particularly in areas such as Islamic finance and governance.

**Figure 15:**
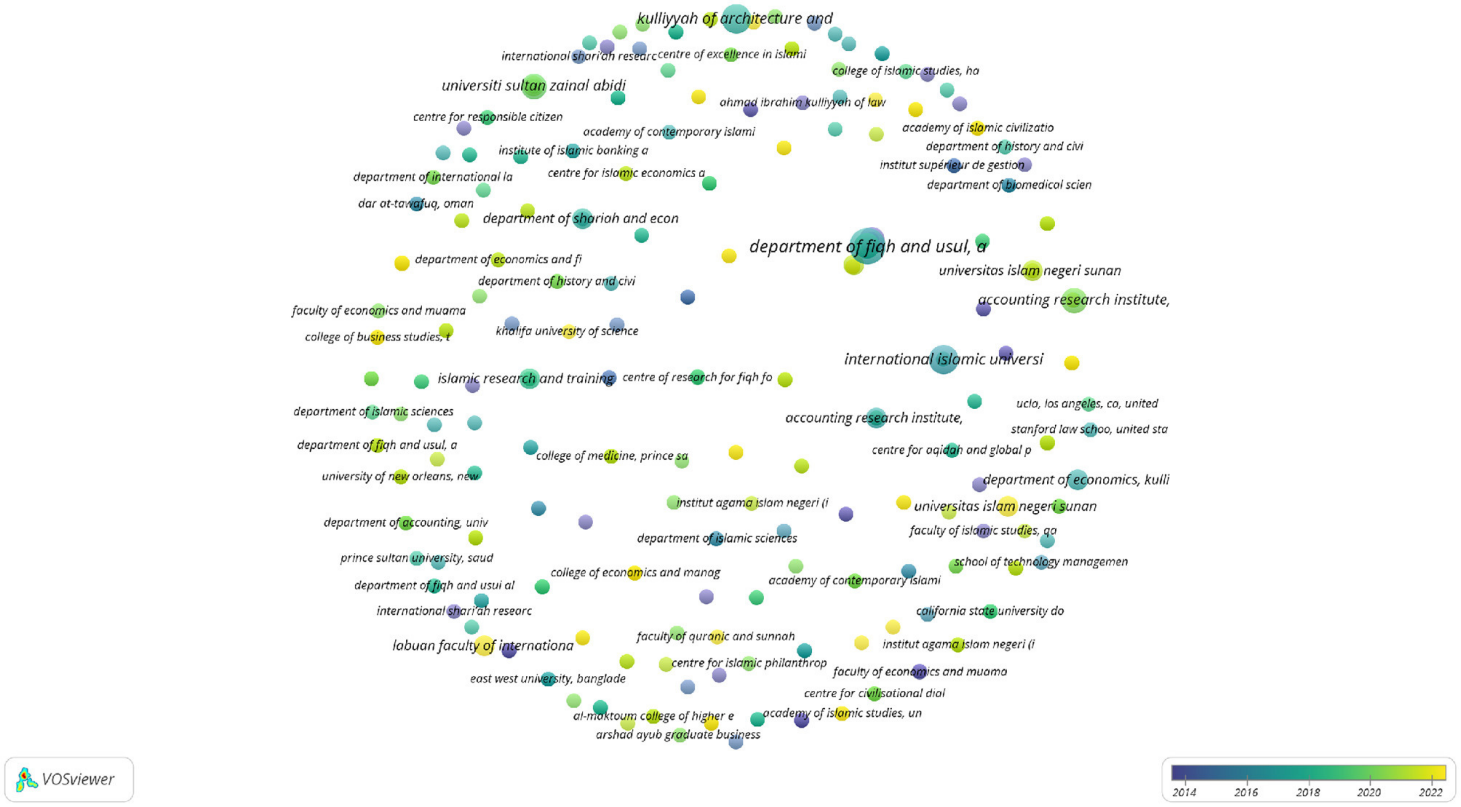
Co-author map of organizations in Scopus.

In Scopus, 354 organizations met the threshold of one document (Figure 15).

The analysis highlights a vibrant and geographically diverse research community engaged in exploring the objectives of Islamic law. The Department of *Fiqh* and *Usul* in the Academy of Islamic Studies at the University of Malaya in Kuala Lumpur stands out as a leading organization in *maqāṣid* research. With six documents and 50 citations, it demonstrates both high productivity and significant impact, supported by a total link strength of 11. This indicates a strong engagement with the research community and substantial contributions to the field.

The Center for Science and Environment Studies at the Institute of Islamic Understanding Malaysia (IKIM), along with the Programme of Applied Science with Islamic Studies at the Academy of Islamic Studies at the University of Malaya, show a focused interest in integrating Islamic studies with other disciplines. The Center for Science and Environment Studies has produced four documents with 25 citations, while the Programme of Applied Science with Islamic Studies has contributed three documents with 25 citations as well. Both centers share a strong link strength of 6, suggesting their collaborative efforts and strong networks within the research community.

Organizations outside of Malaysia, such as Al-Maktoum College of Higher Education in the UK, the College of Islamic Studies at Hamad Bin Khalifa University in Qatar, and the Islamic Economics Institute at King Abdulaziz University in Saudi Arabia, indicate global interest in and diverse approaches to *maqāṣid* research. These institutions, while contributing fewer documents, such as 1 document with 39 citations for Al-Maktoum College and 1 document with 49 citations for King Abdulaziz University, still highlight the international scope and relevance of the field.

Many Indonesian universities, including various branches of Universitas Islam Negeri (UIN), are also involved in *maqāṣid* research. Although their document counts are lower, in some cases they have demonstrated noteworthy link strengths. This suggests a foundational yet growing interest in *maqāṣid* studies in the Indonesian context, which exemplifies the expanding geographic engagement with Islamic legal objectives across Southeast Asia.

### 3.5 Journals publishing on *Maqāṣid* studies

**Figure 16:**
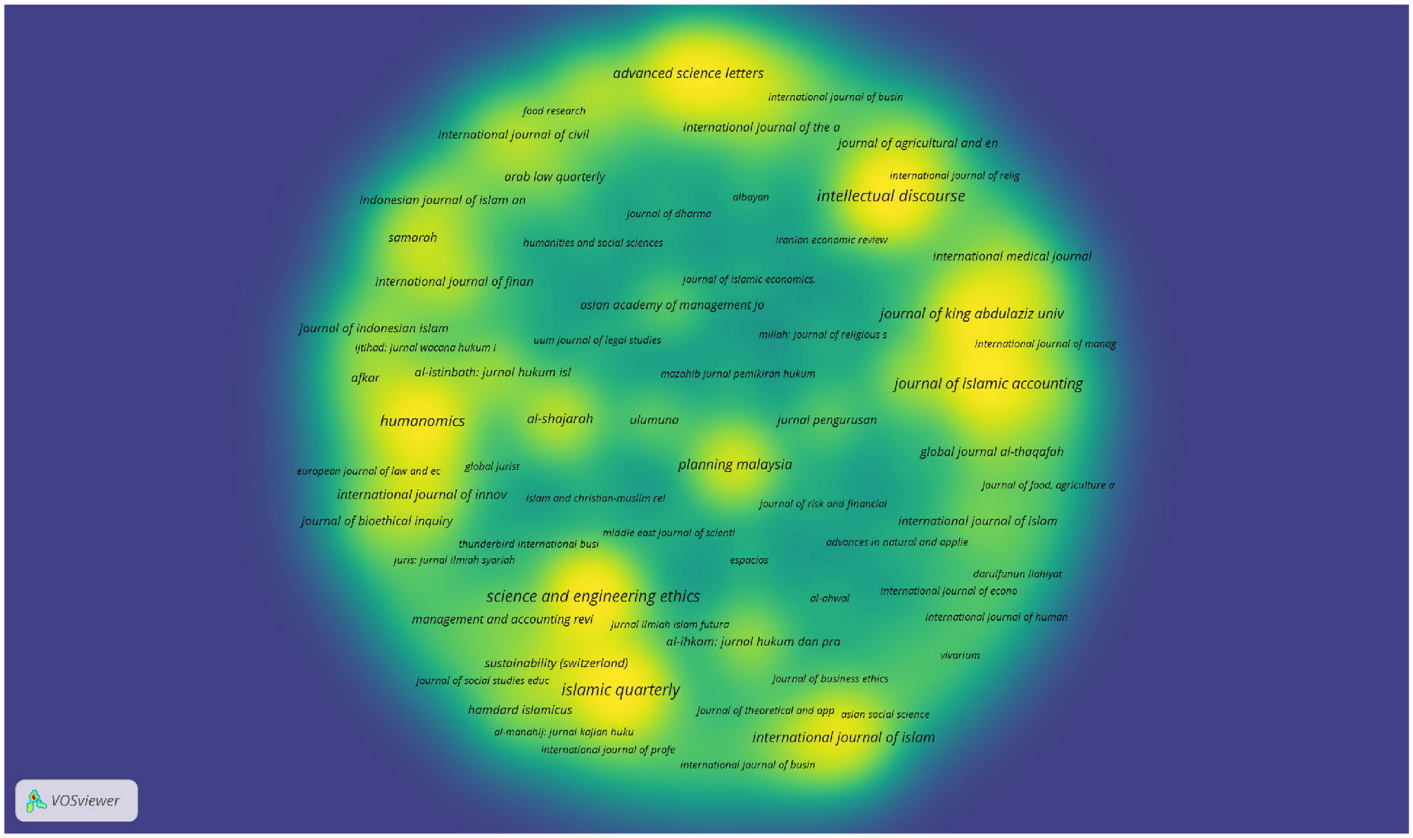
The visualization map of journals publishing in *Maqāṣid* studies in Scopus.

Insofar as sources or journal publications on *maqāṣid* studies are concerned, 99 journals are indexed in Scopus, as shown in Figure 16.

**Figure 17:**
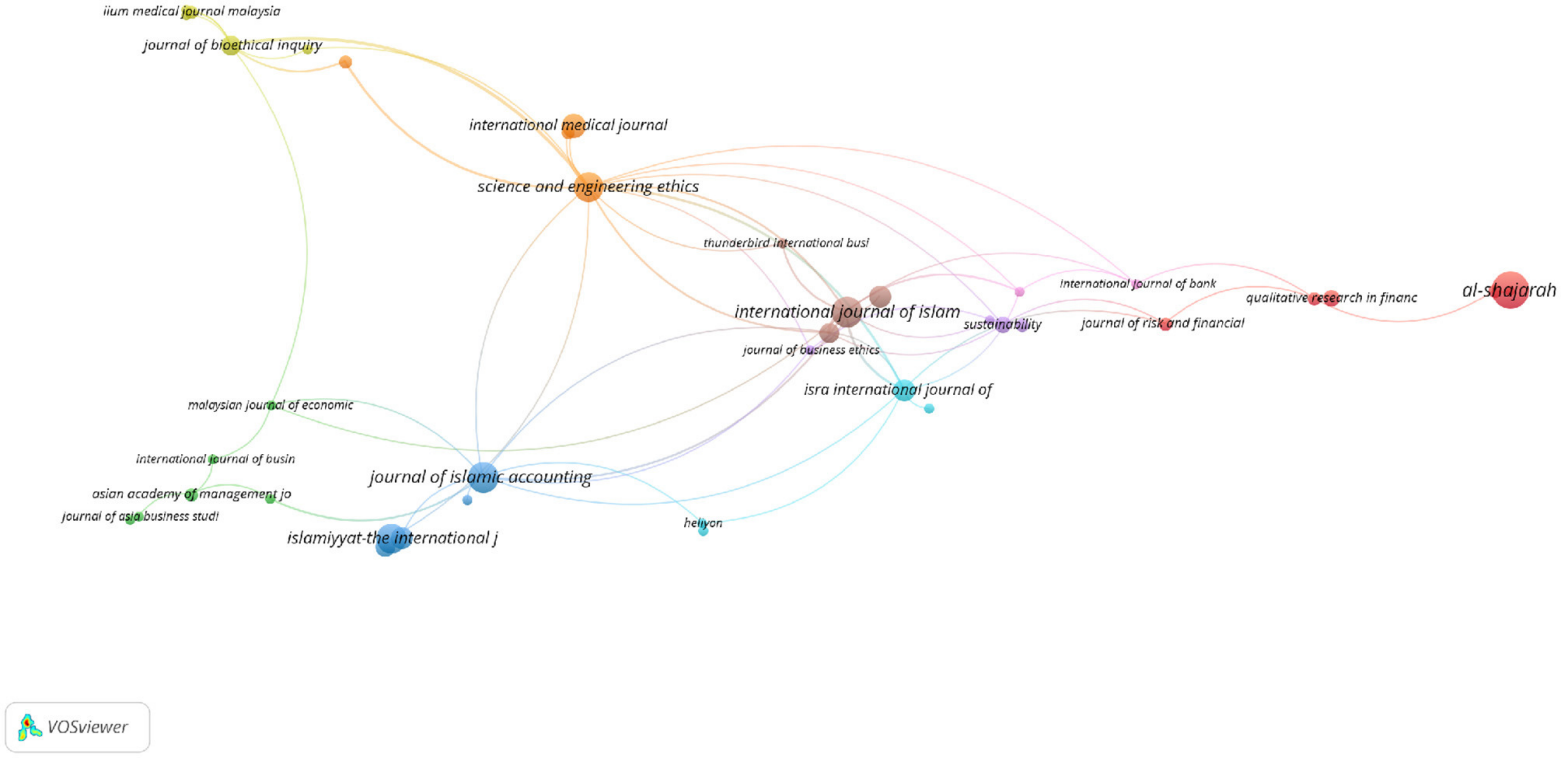
The visualization map of journals publishing in *Maqāṣid* studies in WoS.

By the same token, 92 indexed journals in WoS published *maqāṣid*-related topics, as is obvious in Figure 17. Table 6 presents the top ten journals in terms of the number of publications in both databases.

**Table 6:** The top 10 journals in terms of publications in the two datsets.

**Scopus**	**No**	**Web of science**	**NO**.
Islamic Quarterly	8	Al-Shajarah	15
Intellectual Discourse	8	Journal of Islamic Accounting and Business Research	10
Science and Engineering Ethics	8	International Journal of Islamic and Middle Eastern Finance and Management	10
Humanomics	7	Islamiyyat—The International Journal of Islamic Studies	9
Journal of Islamic Accounting and Business Research	7	Intellectual Discourse	8
Journal of King Abdulaziz University, Islamic Economics	6	Science and Engineering Ethics	8
Advanced Science Letters	6	International Medical Journal Malaysia	6
Planning Malaysia	5	Global Journal Al-Thaqafah	5
International Journal of Islamic and Middle Eastern Finance and Management	5	International Journal of Islamic Thought	5
ISRA International Journal of Islamic Finance	4	International Journal of Ethics and Systems	5

In fact, several journals emerged as key publications in both indexing platforms. This kind of intersection highlights their prominence in *maqāṣid* studies and related fields. For example, *Intellectual Discourse* and *Science and Engineering Ethics* are indexed in both databases and each have 8 publications. The inclusion is a clear indication of their consistent scholarly influence across multiple disciplines. In a similar vein, the *Journal of Islamic Accounting and Business Research* shows a strong presence with seven publications in Scopus and 10 in Web of Science (WoS). This reflects the journal's critical role in the exploration of Islamic accounting practices. Similarly, the *International Journal of Islamic and Middle Eastern Finance and Management* is well-represented, with five publications in Scopus and 10 in Web of Science (WoS). Again, this inclusion underscores its importance in Islamic finance research. *Al-Shajarah* stands out with four publications in Scopus and 15 in Web of Science (WoS), which demonstrates the journal's broad appeal in Islamic thought and civilization studies. Finally, the *Global Journal Al-Thaqafah* maintains a balanced presence with five publications in both datasets, which further illustrates its interdisciplinary impact in cultural and Islamic studies. It is worthwhile to note that the intersections at the journal level do not necessarily translate into intersections at the level of specific authors or article titles. After carefully checking the titles of articles in both datasets, it appears that there are no intersections between the titles in the Scopus and Web of Science (WoS) datasets. This indicates that the same article titles do not appear in both datasets, which clearly suggests that the publications in these journals are distinct across the two datasets.

## 4 Discussion

This scientometric study has attempted to synthesize research in the field of *maqāṣid al-shariī'ah* by employing quantitative metrics to explore trending research topics and survey how they have been investigated over a period of two decades. The findings of this study demonstrate the evolution of *maqāṣid al-shariī'ah* studies. Research outputs in *maqāṣid* studies appear in various disciplines including religion, business and economics, science and technology, and medicine among others. *Maqāṣid al-shariī'ah* as a new paradigm in Islamic law appears to be the major cluster suggesting a focus on the objectives and purposes of Islamic law. Most of the studies tend to present *maqāṣid* as a viable approach of applied Islamic thought that basically aims to ensure the wellbeing of humanity in this world as well as their prosperity in the hereafter. The studies show a focus on *daruriyat* (necessities), including safeguarding life, intellect, progeny, etc. Such findings align with previous studies and systematic reviews (Auda, 2008a; Alias, 2021; Maulida and Ali, 2023) as well as *maqāṣid* studies in other languages such as al-Barghuthi (2021), Muḥammad (2022), and Muṣṭafā (2022).

A strong research interest in the application of *maqāṣid* for tackling financial issues and challenges is also noticed across the two datasets. This finding is in line with those of other studies including (Shinkafi and Ali, 2017; Maulida and Ali, 2023). The prominence of “Malaysia” with a considerable number of occurrences presents it as a hub for *maqāṣid al-shariī'ah* studies especially in Islamic finance and banking studies. This is in agreement with the findings of studies such as Awang et al. (2014), Saoqi (2017), Hashi (2019), Ishak and Asni (2020), and Hamid (2021). The frequent occurrences of keywords such as “Islamic banking,” “Islamic law,” and “Maqasid Al-Shari'ah” suggest a balanced interest in the practical, legal, and ethical dimensions of Islamic jurisprudence as it applies to financial institutions. The integration of *maqāṣid* into Islamic finance, particularly in the Gulf and Southeast Asia is also emphasized in many Arabic-language studies such as Al-Raysuni (2012), Ibn Ṣāliḥ (2023), and Na‘ja and Ibn Zaynab (2023)

Interestingly, “Islamic banks” has a slightly lower count across the two datasets, which could imply that the research is more centered on the broader concepts of Islamic finance and banking than on the institutions themselves. “Human rights” and “COVID-19”, “bio-ethics” and the like have also been featured in the research landscape on *maqāṣid* studies to a lesser extent, which indicate that they are emerging areas of interest or specialized niches within *maqāṣid* studies research (Rane, 2011a; Laluddin, 2012; Delagic and Ghalia, 2018; Azmi, 2022).

Arabic sources have also contributed significantly to this area, with studies from various Arab universities showing the application of *maqāṣid* in medical ethics and public health. Examples of such studies include al-Khaṭīb (2019), Al-Fārisi (2023), Hilāl (2023), and Khalifa (2023).

The dynamic and evolving nature of *maqāṣid al-shariī'ah* attracts the attention of researchers in various fields. The approach has been used to formulate Islamic ethical principles to tackle contemporary societal, medical, and technological issues. This finding is supported by the findings of previous bibliometric studies such as Sholihin et al. (2021) and Maulida and Ali (2023). The dynamism of the field has made *maqāṣid* suitable for addressing global emerging challenges such as COVID-19 and vaccination (Ghazali et al., 2023), artificial intelligence (Mohadi and Tarshany, 2023), peace agreements (Shemer, 2021), hate speech (Mayasari and Cahya, 2021), combating gangs and mafias (Ekşi et al., 2018), sustainable development (Al-Qaṣabi, 2021), Islamic Criminal Legislation (Al-Bashir Khālid, 2024), among others.

## 5 Conclusions

This study examined the development of research related to *maqāṣid al-shariī'ah* in two databases, namely Scopus and Web of Science (WoS) over the past two decades based on a total of 400 documents from both databases. Furthermore, this study employed keyword mapping to delineate topic trends in the field of *maqāṣid al-shariī'ah*. It also introduced thematic maps for density and centrality in the forms of co-keyword network visualizations and co-keyword overlay visualizations. Such analysis provides profound insights into the dynamism of research within the domain of the *maqāṣid al-shariī'ah*.

The development of publications between 2000 and 2022 for the two databases indicates distinct upward trends in cumulative publications and annual growth. Research outputs in *maqāṣid al-shariī'ah* studies span various disciplines including religion, business and economics, science and technology, and medicine among others. The high counts of keywords such as “Islamic finance” indicate a strong research interest in the application of *maqāṣid al-shariī'ah* to tackle financial issues and challenges. A vibrant and diverse global research landscape exists for *maqāṣid al-shariī'ah*, with Malaysia and Indonesia leading in terms of productivity and impact.

This study presents several limitations. As typical of scientometric analyses, this investigation confines its scope to the interval between 2000 and 2022. Consequently, it may have omitted relevant research published outside this timeframe. Moreover, the research was confined to two databases: Scopus and Web of Science (WoS). This is a common limitation in scientometric studies, as it is nearly impossible to cover all available databases comprehensively. The majority of scientometric analyses are typically confined to a single database, such as Scopus or Web of Science (WoS), due to their extensive and reliable coverage of peer-reviewed literature. Although these platforms are internationally recognized for their comprehensive coverage, future studies should broaden the investigative scope to include additional repositories such as Google Scholar.

Another critical limitation is this study's exclusive focus on English-language publications. This restriction likely excludes significant contributions in other languages, notably Arabic, Malay, Bahasa Indonesia, Turkish, and Persian, which may contain substantial scholarly discourse on *maqāṣid al-shariī'ah*. Future research can address this gap by including additional databases such as Google Scholar, which hosts a wider array of publications in multiple languages, including extensive literature in Arabic and other regional languages. Expanding the scope to include such databases would provide a more comprehensive understanding of the global trends in *maqāṣid* studies across linguistic and regional boundaries.

Furthermore, this study predominantly examines journal articles, thereby giving insufficient consideration to other scholarly outputs like books, conference papers, and dissertations. This focus may skew the representation of the research landscape, as these latter formats often offer critical insights and theoretical developments that are not typically found in journal articles. Future research should aim to integrate these diverse sources to provide a more comprehensive analysis of the research landscape in this emerging field of Applied Islamic Thought.

## Data Availability

The original contributions presented in the study are included in the article/supplementary material, further inquiries can be directed to the corresponding author.
